# A Role for ATF2 in Regulating MITF and Melanoma Development

**DOI:** 10.1371/journal.pgen.1001258

**Published:** 2010-12-23

**Authors:** Meera Shah, Anindita Bhoumik, Vikas Goel, Antimone Dewing, Wolfgang Breitwieser, Harriet Kluger, Stan Krajewski, Maryla Krajewska, Jason DeHart, Eric Lau, David M. Kallenberg, Hyeongnam Jeong, Alexey Eroshkin, Dorothy C. Bennett, Lynda Chin, Marcus Bosenberg, Nic Jones, Ze'ev A. Ronai

**Affiliations:** 1 Sanford-Burnham Medical Research Institute, La Jolla, California, United States of America; 2 Paterson Institute for Cancer Research, University of Manchester, Manchester, United Kingdom; 3 Department of Medicine, Yale University, New Haven, Connecticut, United States of America; 4 Basic Medical Sciences, St. George's, University of London, London, United Kingdom; 5 Department of Medical Oncology, Dana-Farber Cancer Institute, Boston, Massachusetts, United States of America; 6 Department of Pathology Yale University, New Haven, Connecticut, United States of America; Stanford University School of Medicine, United States of America

## Abstract

The transcription factor ATF2 has been shown to attenuate melanoma susceptibility to apoptosis and to promote its ability to form tumors in xenograft models. To directly assess ATF2's role in melanoma development, we crossed a mouse melanoma model (*Nras^Q61K^::Ink4a^−/−^*) with mice expressing a transcriptionally inactive form of ATF2 in melanocytes. In contrast to 7/21 of the *Nras^Q61K^::Ink4a^−/−^* mice, only 1/21 mice expressing mutant ATF2 in melanocytes developed melanoma. Gene expression profiling identified higher MITF expression in primary melanocytes expressing transcriptionally inactive ATF2. MITF downregulation by ATF2 was confirmed in the skin of *Atf2^−/−^* mice, in primary human melanocytes, and in 50% of human melanoma cell lines. Inhibition of MITF transcription by MITF was shown to be mediated by ATF2-JunB–dependent suppression of SOX10 transcription. Remarkably, oncogenic BRAF (V600E)–dependent focus formation of melanocytes on soft agar was inhibited by ATF2 knockdown and partially rescued upon shMITF co-expression. On melanoma tissue microarrays, a high nuclear ATF2 to MITF ratio in primary specimens was associated with metastatic disease and poor prognosis. Our findings establish the importance of transcriptionally active ATF2 in melanoma development through fine-tuning of MITF expression.

## Author Summary

Understanding mechanisms underlying early stages in melanoma development is of major interest and importance. Recent studies indicate a role for MITF, a master regulator of melanocyte development and biogenesis, in melanoma progression. Here we demonstrate that the transcription factor ATF2 negatively regulates MITF transcription in melanocytes and in about 50% of melanoma cell lines. Increased MITF expression, seen upon inhibition of ATF2, effectively attenuated the ability of BRAF^V600E^-expressing melanocytes to exhibit a transformed phenotype, an effect partially rescued when MITF expression was also blocked. Significantly, the development of melanoma in mice carrying genetic changes seen in human tumors was inhibited upon inactivation of ATF2 in melanocytes. Melanocytes from mice lacking active ATF2 expressed increased levels of MITF, confirming that ATF2 negatively regulates MITF and implicating this newly discovered regulatory link in melanoma development. Primary melanoma specimens that exhibit a high nuclear ATF2-to-MITF ratio were found to be associated with metastatic disease and poor prognosis, further substantiating the significance of MITF control by ATF2. In all, these findings provide genetic evidence for the role of ATF2 in melanoma development and indicate an ATF2 function in fine-tuning MITF expression, which is central to understanding MITF control at the early phases of melanocyte transformation.

## Introduction

Malignant melanoma is one of the most highly invasive and metastatic tumors [1], and its incidence has been increasing at a higher rate than other cancers in recent years [2]. Significant advances in understanding melanoma biology have been made over the past few years, thanks to identification of genetic changes along the MAPK signaling pathway. Those include mutations in *BRAF*, *NRAS, KIT and GNAQ,* all of which result in a constitutively active MAPK pathway [3]–[5]. Consequently, corresponding transcription factor targets such as microphthalmia-associated transcription factor (MITF) [6], AP2 [7], and C-JUN [8] and its heterodimeric partner ATF2 [9] are activated and induce changes in cellular growth, motility and resistance to external stress [10], [11]. In addition, constitutively active MAPK/ERK causes rewiring of other signaling pathways [4]. Among examples of rewired signaling is upregulation of C-JUN expression and activity [8], which potentiates other pathways, including PDK1, AKT and PKC, and plays a critical role in melanoma development [12].

Activating transcription factor 2 (ATF2), a member of the bZIP family, is activated by stress kinases including JNK and p38 and is implicated in transcriptional regulation of immediate early genes regulating stress and DNA damage responses [13]–[15] and expression of cell cycle control proteins [16]. To activate transcription, ATF2 heterodimerizes with bZIP proteins, including C-JUN and CREB [17], [18], both of which are constitutively upregulated in melanomas [8]. ATF2 is also implicated in the DNA damage response through phosphorylation by ATM/ATR [19]. Knock-in mice expressing a form of ATF2 that cannot be phosphorylated by ATM are more susceptible to tumor development [20]. Nuclear localization of ATF2 in melanoma tumor cells is associated with poor prognosis [21], likely due to transcriptional activity of constitutively active ATF2. Indeed, expression of transcriptionally inactive ATF2 or peptides that attenuate endogenous ATF2 activity inhibits melanoma development and progression in xenograft models [22]–[26]. These studies suggest that ATF2 is required for melanoma development and progression.

The transcription factor MITF has been shown to play a central role in melanocyte biology and in melanoma progression [27], [28]. Yet, the role of MITF in early stages of melanoma development remains largely unexplored. Factors controlling MITF transcription have been well documented and include transcriptional activators, such as SOX10, CREB, PAX3, lymphoid enhancer-binding factor 1 (LEF1, also known as TCF), onecut domain 2 (ONECUT-2) and MITF itself [29]–[33], as well as factors that repress MITF transcription, including BRN2 and FOXD3 [34], [35]. In addition, MITF is subject to several post translational modifications which affect its availability and activity, including acetylation, sumoylation and ubiquitination [27], [28].

To directly assess the importance of ATF2 in melanoma development, we employed a mouse melanoma model in which ATF2 is selectively inactivated in melanocytes. We demonstrate that melanoma development is markedly attenuated in mice expressing a transcriptionally inactive form of ATF2 in melanocytes. Surprisingly, ATF2 control of melanoma development was mediated, in part, through its negative regulation of SOX10 and consequently of MITF transcription. Inhibition of ATF2 abolished mutant BRAF-expressing melanocytes' ability to form foci on soft agar, which was partially rescued when expression of MITF was attenuated. The significance of these findings is underscored by our observation of human melanoma tumors, in which high ratio of nuclear ATF2 to MITF expression was associated with poor prognosis. These findings identify a novel mechanism underlying melanocyte transformation and melanoma development.

## Results

### Generation of melanocyte-specific *ATF2* mutant mice

**Figure 1:**
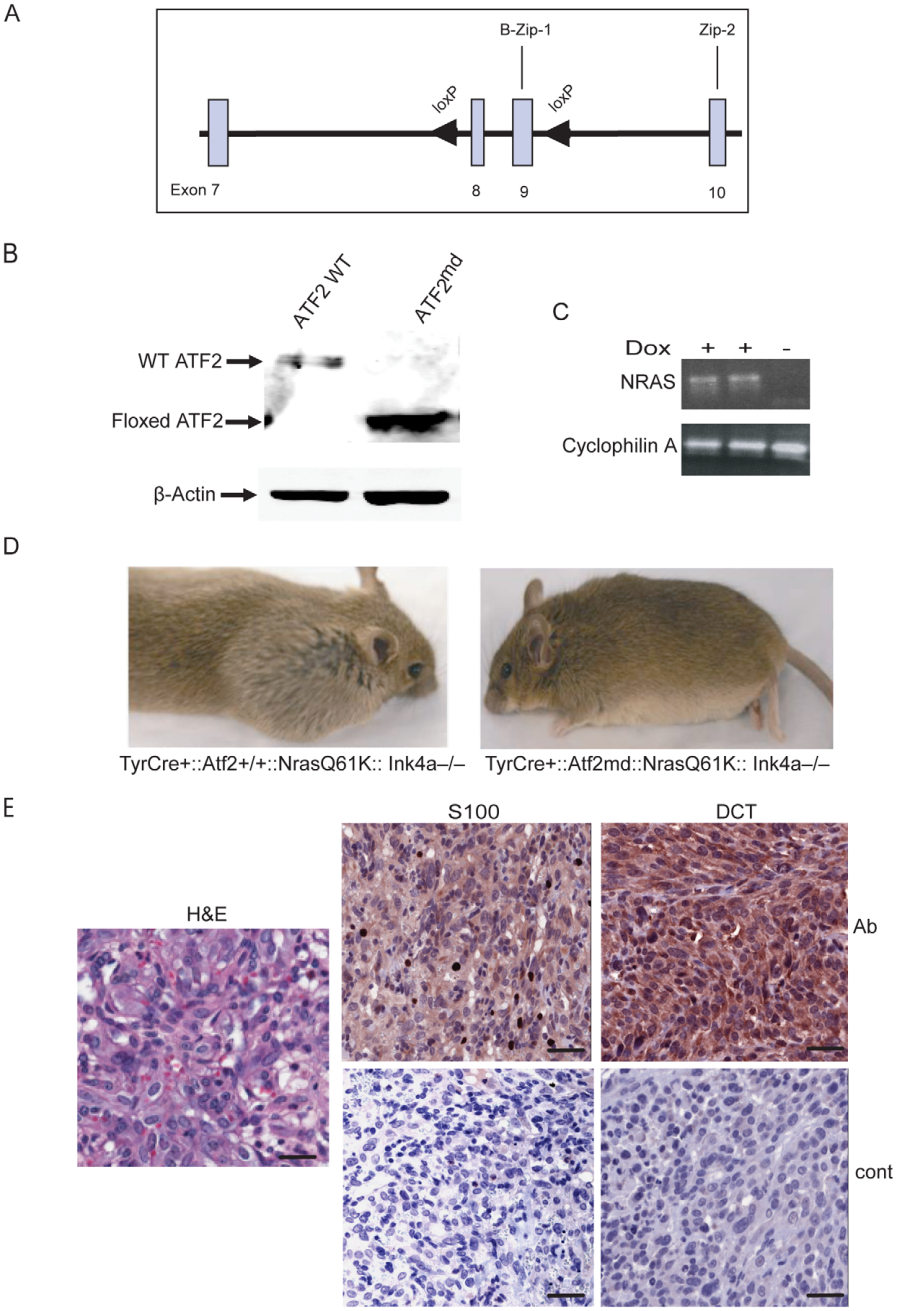
Melanoma development is inhibited in *Nras/Ink4a* mice expressing a melanocyte-specific ATF2 mutation. A. Targeting strategy shows wild-type allele of *Atf2* encompassing exons 8 and 9 (boxes) and flanking loxP sequences (arrowheads). B. Expression of ATF2 assessed by immunoblot in melanocytes derived from *TyrCre^+^*::*Atf2^+/+^::Nras^Q61K^::Ink4a^−/−^* and *TyrCre^+^*::*Atf2^md^::Nras^Q61K^::Ink4a^−/−^* mice and treated with 4-OHT, enabling expression of a mutant lacking the DNA binding and leucine zipper domains. β-actin served as a loading control. C. Induction of mutant NRAS in melanocytes. Shown is RT-PCR analysis (20 cycles) of NRAS^Q61K^ transcript levels in melanocyte cultures treated with doxycycline (2mg/ml). RNA from untreated melanocytes served as a control (right lane), and Cyclophilin A served as an internal control. D. Melanoma development is inhibited in *TyrCre^+^::Atf2^md^::Nras^Q61K^::Ink4a^−/−^* mice. Image represents animals of *TyrCre^+^*::*Atf2^+/+^::Nras^Q61K^::Ink4a^−/−^* or *TyrCre^+^*::*Atf2^md^::Nras^Q61K^::Ink4a^−/−^* genotype that were analyzed for tumor formation within 8–32 weeks. E. Representative staining of melanoma tumors from mice of the *TyrCre^+^*::*Atf2^+/+^Nras^Q61K^Ink4a^−/−^*genotype for melanoma markers. Immunohistochemistry was performed on 5 µM paraffin-embedded samples. Sections were incubated with S-100 and DCT antibody (Ab) or control secondary antibody (Cont) and counterstained with hematoxylin. Scale bar = 50 micron. Slides were scanned by scanscope at 20× (IHC) and 40× (H&E).

Global *Atf2* knockout in mice leads to early post-natal death [36]. Therefore, the Cre-loxP system was utilized to disrupt *Atf2* in melanocytes. Deletion of its DNA binding domain and a portion of the leucine zipper motif results in a transcriptionally inactive form of ATF2 (Figure 1a; [36]). To generate loss-of-function mutants, we established mice that would allow CRE-dependent deletion of these domains. Mice homozygous for the loxP-flanked (floxed) *Atf2* gene (*Atf2^f/f^*) were born at the expected Mendelian ratios and presented no apparent abnormalities. In addition, in several tissues analyzed, ATF2 expression levels were comparable between *WT* and *Atf2^f/f^* mice (data not shown).

To elucidate the role of ATF2 in melanoma, *Atf2^f/f^* mice were crossed with mice harboring a 4-hydroxytamoxifen (OHT)-inducible Cre recombinase-estrogen receptor fusion transgene under the control of the melanocyte-specific tyrosinase promoter, designated *Tyr::Cre^ER^(T2)*. Upon administration of OHT, we predicted that CRE-mediated recombination would be induced in a spatially and temporally controlled manner in embryonic melanoblasts, melanocytes, and in putative melanocyte stem cells [37]. The resulting *Atf2^f/f^/Tyr-Cre^ER^(T2)* mice, designated melanocyte-deleted (md) *Atf2^md^*), indeed expressed the gene encoding the ATF2 transcriptional mutant in melanocytes. Immunoblot analysis of ATF2 protein confirmed that melanocytes prepared from wild-type TyrCre^+^ *::Atf2* ^+/+^ *::Nras* ^+^ *::Ink4a^−/−^* (WT) mice express a 70 kDa band corresponding to full length ATF2, whereas melanocytes of TyrCre^+^ *::Atf2* ^md^ *::Nras* ^+^ *::Ink4a^−/−^* mice express only a 55 kDa band, corresponding to the size of ATF2 lacking the DNA binding and leucine zipper domains (Figure 1b).

### Disruption of ATF2 in melanocytes inhibits melanoma formation

To address the role of ATF2 in *de novo* melanoma formation *Tyr::Cre^ER^::Nras^Q61K^::Ink4a^−/−^* (KO of exon 2–3 of *Cdkn2a* locus, which encodes for both p16^Ink4a^ and p19^Arf^; [38]) mice, which develop spontaneous melanoma (Lynda Chin, unpublished observations), were crossed with *Atf2^md^* mice. Similar to findings reported by Ackermann et al. [39], mutant *N-Ras/Ink4a^−/−^* mice developed melanoma within 8–12 weeks with metastatic lesions often seen in the lymph nodes. However, the incidence of melanoma was lower in *Tyr::Cre^ER^::Nras^Q61K^::Ink4a^−/−^* mice used in the present study (50% penetrance, of which 50% of the tumors were confirmed to be melanoma), probably because expression of mutant *NRAS* was induced only after birth, as opposed to activation of NRAS during embryogenesis, as reported in [39]). Thus, *Atf2^md^::N-Ras^Q61K^::Ink4a^−/−^* mice were used to assess changes in melanoma incidence in the absence of functional ATF2 over a period of up to 8 months. In all cases, mouse skin was treated with Tamoxifen within 3–5 days after birth to inactivate ATF2 (Figure 1b) and with doxycycline in their drinking water to induce expression of the NRAS mutant transgene (See Materials and Methods for details; Figure 1c). In the control group (*Tyr::Cre^ER^::Atf2^+/+^::Nras^Q61K^::Ink4a^−/−^*), 11/21 mice (52%) developed tumors within 8–16 weeks (Table 1). In ATF2 heterozygotes (*Tyr::Cre^ER^::Atf2^−/+^::Nras^Q61K^::Ink4a^−/−^*), 18/44 mice (41%) developed tumors within 8–16 weeks, and in the *Tyr::Cre^ER^::Atf2^md^::Nras^Q61K^::Ink4a^−/−^* group only 3 of 21 animals (15%) developed tumors within 24–36 weeks (Figure 1d and Table 1). To evaluate tumor type, we examined melanoma markers including DCT and S100 in all tumors (Figure 1e, Figure S1). This analysis identified 55–63% of tumors as melanomas in both the *Atf2^+/+^* (7/11) and *Atf2^+/−^* (10/18) groups (Table 2). Only one of the three tumors observed in the *Atf2^md^* group was identified as a melanoma. Kaplan Meier curve did not reveal significant differences in survival among the different genotypes, probably since this study was primarily designed to follow tumor incidence. Common to all genotypes, most tumors that were not identified as melanomas were fibrosarcomas and lymphomas, consistent with previous reports [38]. These data suggest that transcriptionally active ATF2 is required for melanoma development in the *Nras^Q61K^::Ink4a^−/−^* mouse melanoma model.

**Table 1:** Tumor incidence in mixed genetic backgrounds.

Genotype	No of mice	Tumor Incidence
*ATF2* ^+/+^TyrCre^+^ *Nras* ^+^ *Ink4a^−/−^*	21	11/21 (52%)
*ATF2* ^+/−^TyrCre^+^ *Nras* ^+^ *Ink4a^−/−^*	44	18/44 (41%)
*ATF2* ^−/−^TyrCre^+^ *Nras* ^+^ *Ink4a^−/−^*	21	3/21 (15%)

Shown is the tumor incidence in the different genotypes.

**Table 2:** Tumors positive for melanoma markers S100 and DCT.

Genotype	Melanomas	Non melanomas
*ATF2* ^+/+^TyrCre^+^ *Nras* ^+^ *Ink4a^−/−^*	7/21 (33%)	4/21 (19%)
*ATF2* ^+/−^TyrCre^+^ *Nras* ^+^ *Ink4a^−/−^*	10/44 (22%)	8/44 (18%)
*ATF2* ^−/−^TyrCre^+^ *Nras* ^+^ *Ink4a^−/−^*	1/21 (5%)	2/21 (10%)

In order to distinguish melanoma from non-melanoma tumors were subjected to staining with S100 and DCT (see Figure 1E, Figure S1). Only those stained positive for both markers were considered as melanomas. Number of positive stained out of total melanomas is shown (percent positive) for each of the genotypes. Statistical analysis using Chi-square assay revealed that the differences between # of melanomas between the ATF2^−/−^ and ATF2^+/+^ genotypes are significant (p = 0.018). Differences among the other groups were not significant (differences between # of melanomas between the ATF2^−/+^ and ATF2^+/+^ p = 0.36; differences between # of non-melanomas between the ATF2^−/−^ and ATF2^+/+^ groups p = 0.37. differences between # of non-melanomas between the ATF2^−/−^ and ATF2^−/+^ p = 0.92).

### Identification of MITF as an ATF2-regulated gene

To assess the mechanism underlying ATF2's contribution to melanoma development, we conducted gene profiling array analysis of primary melanocytes prepared from *Tyr::Cre^+^::Atf2^+/+^::Nras^Q61K^::Ink4a^−/−^* and *Tyr::Cre^+^::Atf2^md^::Nras^Q61K^::Ink4a^−/−^* mice. Analysis was limited to melanocytes, since, as reported above, only one melanoma formed in the ATF2 mutant group. In all cases, ATF2 was inactivated and NRAS was induced in culture within 48h of plating cells, as monitored by western blots (Figure 1b, 1c and data not shown). Melanocytes were enriched, and immunostaining with appropriate markers confirmed that samples were free of keratinocytes and fibroblasts (data not shown; see Materials and Methods for details). RNA was prepared from cultures and two biological and technical replicates were used for data analysis. As shown in Table 3, among transcripts differentially expressed in ATF2 WT and mutant cultures were several factors that play an important role in melanocyte pigmentation, including *Mitf, Silver, Tyrp1* and *Dct*. qPCR analysis, performed on independently prepared RNA samples from melanocytes expressing WT (*Tyr::Cre^+^::Atf2^+/+^::Nras^Q61K^::Ink4a^−/−^*) or mutant ATF2 (*Tyr::Cre^+^::Atf2^md^::Nras^Q61K^::Ink4a^−/−^*), confirmed altered expression of pigmentation genes (Table 3). These data provide the initial indication that ATF2 negatively regulates *Mitf* and several other important pigmentation genes. As the pigmentation genes identified in this array are known to be regulated by MITF [27], we focused on regulation of MITF by ATF2.

**Table 3:** Microarray analysis of *ATF2^+/+^TyrCre^+^Nras^+^Ink4a^−/−^* and *ATF2^−/−^TyrCre^+^Nras^+^Ink4a^−/−^*melanocytes and confirmation by qPCR.

Gene name	Microarray (ATF2^−/−^/WT)	qPCR confirmation
Cyclin D1	0.4	0.425
ESAM1	0.09	0.123
Angiopoietin 2	0.254	0.129
KIflc	0.419	0.56
MITF	7.3	1.91
PCDH7	0.46	0.225
Silver	45.27	74.88
DCT	9.0	18.9
Tyrp1	2.3	12.7
Tgfbi	0.34	0.49

Primary melanocytes were isolated and inactivation of ATF2+ induction of N-Ras expression was performed in culture (see Materials and Methods for details). RNA prepared from the cells was used for array analysis. Data shown represents 2 biological and 2 technical replicates for the array studies and 3 replicates for the qPCR analyses (see Materials and Methods for details).

### MITF is negatively regulated by ATF2 in mouse and human melanocytes

**Figure 2:**
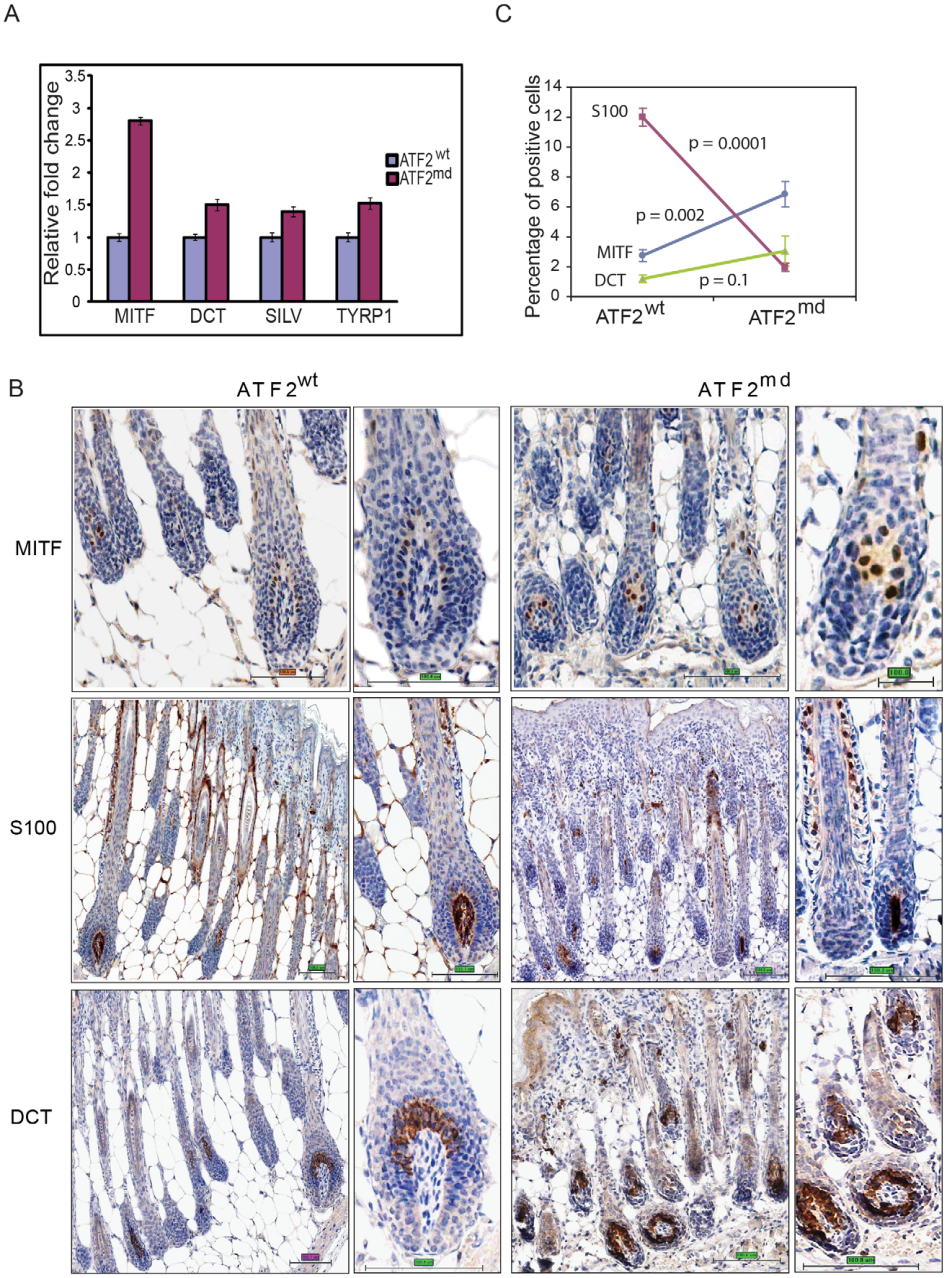
ATF2 negatively regulates MITF in melanocytes. A. qPCR performed on RNA prepared from skin of 4-day-old ATF2 WT or *Atf2^md^* mice (3 mice per sample; see Materials and Methods for details). Shown is representative analysis of 3 independent samples. B. IHC analysis of mouse skin prepared from 4-day-old mice. Staining with corresponding antibodies was performed as indicated in Experimental Procedures. Magnifications shown are 20 and 40×. S100 staining reveals changes in the hair matrix (low magnification) and migrating melanoblasts (higher magnification) Scale bar = 100 micron. C. Quantification of immunostaining was performed using the automated Aperio ScanScope CS system (see Materials and Methods for details). The percentage (%) reflects the amount of positive signal in five selected fields representing longitudinal sections through the skin and containing the entire length of hairs, from the bulb with the subcutis to the epidermis.

To confirm that ATF2 negatively regulates *Mitf* expression, we assessed MITF transcription in primary mouse melanocytes harboring WT (*Tyr::Cre^−^::Atf2^+/+^*) or mutant (*Tyr::Cre^+^::Atf2^md^*) forms of ATF2. RNA prepared from whole skin of these mice (3 mice per group) was subjected qPCR analysis. Significantly, *Mitf* expression was inversely correlated to the presence of functional ATF2; samples obtained from ATF2 mutant skin exhibited a greater than 2-fold increase in MITF expression compared with those obtained from WT ATF2 mice (Figure 2a). Likewise, we found that genes transcriptionally regulated by MITF, such as *Dct*, *Silver* and *Tyrp1*, were upregulated in the skin of mutant ATF2 mice (Figure 2a). The degree of altered expression of pigmentation genes was less pronounced in whole skin samples than in cultured melanocytes (Table 3), probably due to confounding effects of in vitro cell culture. To confirm the qPCR data, we performed immunostaining of skin tissue samples obtained from 4 days old WT or ATF2 mutant mice and observed increased MITF expression in melanocytes from *Atf2^md^* mice relative to their WT counterparts (Figure 2b). Quantification of MITF staining revealed an approximate 2-fold increase in nuclear MITF expression in *Atf2^md^* compared to WT mice (Figure 2c). Of note, the level of S100 staining in the hair matrix was markedly reduced in the skin of *Atf2^md^* mice. At a later time point (2 weeks) representing an advanced stage of melanocyte development, S100 staining was similar in both genotypes, while MITF expression remained upregulated in *Atf2^md^* mice (not shown). In all, these data confirm our initial observations in primary mouse melanocytes that MITF levels are elevated in ATF2 mutant-expressing cells.

**Figure 3:**
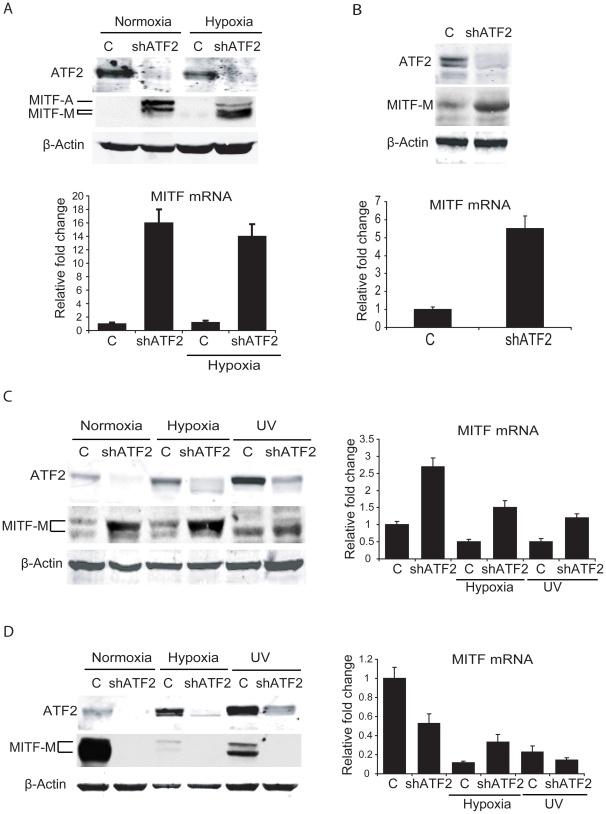
ATF2 regulates MITF in melanocytes and melanoma cells. A. *Upper panel*, ATF2 was knocked down in primary human melanocytes (one 10 cm plate each, 50% confluent) and cells were either untreated or kept under hypoxia (1%) for 16h. Cells were lysed and Western blotting was carried out with the indicated antibodies. *Lower panel*, RNA was extracted from the above samples and qPCR was carried out using MITF primers. Cyclophilin A was served as an internal control. B. *Upper panel*, ATF2 was knocked down in *melan-Ink4a-Arf1* mouse melanocytes and Western blotting was performed using the indicated antibodies. *Lower panel*, qPCR of the above samples was carried out using MITF primers. C. *Left panel*, Lu1205 melanoma cells (one 10 cm plate each, 50% confluent) were transduced with either empty or shATF2 lentiviral vectors. Cells were left untreated, kept under hypoxia (1%), or treated with UV-B (21 mJ/cm^2^ for 6 h) irradiation. Proteins were prepared 6h after UV-B treatment or 16h after growth under hypoxia. Proteins were immunoblotted using the indicated antibodies. *Right panel*, RNA was extracted from cells maintained under the same conditions, and qPCR was performed using MITF primers. Cyclophilin A was served as an internal control for qPCR. D. The experiment was carried out in MeWo melanoma cells as described in panel C.

Additional assessment was performed in *melan*-*Ink4a-Arf1* melanocytes, a line derived from black *Ink4a-Arf* null mice [40], and in primary human melanocytes. In both, ATF2 expression was inhibited by viral infection with the corresponding mouse or human shRNA (shATF2). Infection of either primary human (Figure 3a) or *melan*-*Ink4a-Arf1* melanocytes (Figure 3b) with shATF2 markedly increased MITF transcription and protein expression (Figure 3a, 3b). These findings show that loss of transcriptionally active ATF2 allows higher expression of MITF and strongly suggest that ATF2 negatively regulates MITF expression in melanocytes.

### MITF transcription is negatively regulated by ATF2 in about 50% of human melanoma cells

Given that ATF2 negatively regulates MITF in melanocytes of mouse and human tissues and in related melanocyte cell lines, we asked whether ATF2 also regulates MITF in human melanoma cells. Initially, we assessed changes in MITF expression in six human melanoma lines harboring oncogenic mutations in *BRAF* or *NRAS*, and in which ATF2 expression was effectively inhibited by corresponding shRNA (shATF2). In all cases, shRNA specificity was confirmed using three independent sequences (data not shown). Surprisingly, the six melanoma lines fell into two classes based on distinct patterns of regulation of MITF by ATF2 (Table 4). The first class comprised four of the six melanoma cultures (1205Lu, WM35, WM793 and WM1361), in which MITF expression was elevated 3–6-fold following inhibition of ATF2 expression (Figure 3c, S2a). Conversely, a second class of cells, including MeWo and 501Mel cells, exhibited decreased MITF expression after ATF2 knockdown (KD), suggesting positive regulation of MITF by ATF2 (Figure 3d, S2b). Notably, this latter group showed high levels of basal MITF expression [41], [42], suggesting that regulation of MITF expression in these cells differs mechanistically from that of the first group. Further, in response to stress (UV or hypoxia) the MeWo and 501Mel lines further reduced MITF expression (Figure 3d and data not shown), providing further evidence for differential regulation of MITF in these cells both prior to and in response to stress stimuli. Additional analyses were performed, employing 12 more melanoma cell lines. Inhibition of ATF2 expression revealed that 4/12 exhibited increase in MITF expression, while 6/12 decreased MITF expression. Two of the 12 lines did not exhibit change in MITF expression following ATF2 KD (Table 4, S5). Collectively, out of 18 melanoma lines we found that 8 (44%) retained similar negative regulation of MITF by ATF2 as observed in the melanocytes. However, another 8 (44%) exhibited positive regulation of MITF by ATF2, pointing to a transcriptional switch that occurred in the course of melanocyte transformation. MITF was not affected by altered ATF2 expression in 2/18 cell lines (Table 4, Figure S5). In all, in about 50% of the melanoma cell lines ATF2 elicits negative regulation of MITF, similar to what was seen in human and mouse melanocytes.

**Table 4:** MITF and SOX10 mRNA levels in melanocytes and melanoma cell lines following ATF2 KD.

Melanocytes/Melanoma cells	Relative change in MITF mRNA upon ATF2 KD*	Level of SOX10 upon ATF2 KD**
Hermes 3AMelanocytes	6–7 fold increase	6–8 fold increase
Melan-Ink4a-Arf1Melanocytes	4–6 fold increase	ND
Lu1205	2–3 fold increase	unchanged
WM35	2.5–3.5 fold increase	unchanged
WM1361	4–6 fold increase	3–3.5 fold increase
WM793	1.5–2 fold increase	1.5–2 fold increase
SbCl2	1.5 fold increase	1.5 fold increase
WM4	2 fold increase	2 fold increase
WM9	2 fold increase	1.5 fold increase
WM1650	1.7 fold increase	1.5 fold increase
WM1552	2 fold decrease	unchanged
WM3629	2.5 fold decrease	1.7 fold decrease
501MEL	2.5–3 fold decrease	0.6 fold decrease
SK-MEL-2	2.5 fold decrease	2 fold decrease
SK-MEL-5	3 fold decrease	2 fold decrease
SK-MEL-8	2.5 fold decrease	1.5 fold decrease
MeWO	2–2.5 fold decrease	0.5 fold decrease
UACC903	4 fold decrease	1.5 fold decrease
A2068	unchanged	2.5 fold decrease
WM1366	unchanged	2.3 fold increase

*Relative changes in MITF upon ATF2 KD are shown as fold change at RNA level, determined by qPCR. Data shown represent triplicate analyses.

**Relative changes in SOX10 upon ATF2 knock down (KD) are also quantified by qPCR analysis. ND: not determined.

### ATF2 regulation of MITF is mediated by discrete promoter elements

**Figure 4:**
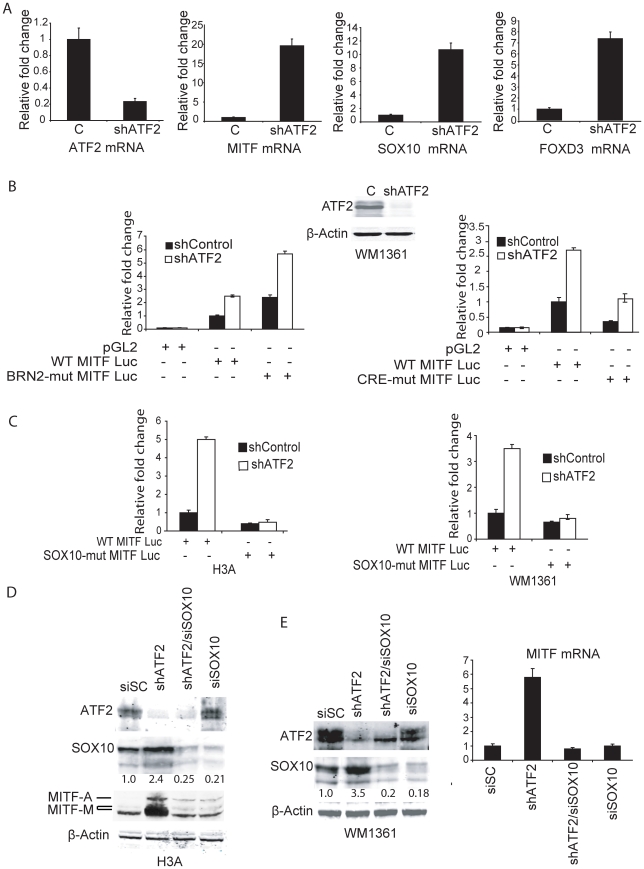
Negative regulation of MITF by ATF2 is mediated by SOX10. A. ATF2 was knocked down in human melanocytes (Hermes 3A) and qPCR was carried out using primers for the indicated mRNAs. B. Melanoma cells (WM1361, one 10 cm plate each, 50% confluent) were transduced with either empty or shATF2 lentiviral particles. Cells were selected for 3 days with puromycin treatment (1.5 µg/ml) and then transfected with either a WT MITF-luciferase reporter or one with a mutant BRN2 site (0.5 µg) along with β-gal (0.1 µg) as an internal control. β-gal activity was normalized for every sample and the relative fold change in reporter activity for control and shATF2 cells is shown. Right Panel- Analysis was performed in WM1361 melanoma cells using a WT or CRE-mutated MITF promoter. C. Analysis was performed as in panel B using human melanocytes H3A (*left panel*) or 1361 melanoma cells (*right panel*) and the WT MITF promoter or a construct in which the SOX10 site was mutated. D. Human melanocytes (H3A; one 10 cm plate each, 50% confluent) were infected with control shRNA (SiSC) or shATF2. After puromycin selection for 3 days, control shRNA cells and ATF2 knock down cells (2×10^6^) were transfected with either scrambled siRNA (SiSC) or siRNA against SOX10. After 72 h, Western analysis was carried out using 50 µg of proteins and the indicated antibodies. E. The same analysis was performed in human melanoma cells. *Right panel*, RNA was extracted from cells and used for qPCR analysis of MITF transcripts.

MITF transcription is regulated by complex positive and negative cues [27]. For instance, while CREB and SOX10 positively regulate MITF, BRN2 and FOXD3 have been shown to downregulate MITF expression [29], [30], [34], [35]. Hence we used melanocytes and representative melanoma lines to assess mechanisms underlying positive or negative regulation of MITF. Infection of the human melanocyte line *Hermes 3A* with shATF2 effectively inhibited ATF2 expression, upregulated *Mitf* transcription and increased transcription of SOX10 and FOXD3 (from 7- to 10-fold) and to a lesser extent of Pax3 and Brn2 (from 1.5- to 2-fold) (Figure 4a, S3a). Similarly, inhibition of ATF2 transcription in human melanoma 1361 cells increased SOX10 and FOXD3 transcription, albeit, to a lesser degree (3- and 1.5-fold, respectively) compared with human melanocytes (Figure S3b). Neither BRN2 nor PAX3 transcription was elevated in melanoma cells in which ATF2 expression was inhibited (Figure S3a). These observations suggest a role for ATF2 in FOXD3- and SOX10-mediated regulation of MITF transcription in melanocytes and melanoma cells.

To assess the possible role of FOXD3 in regulation of MITF we inhibited FOXD3 expression in melanocytes expressing control shRNA and shATF2. Inhibition of FOXD3 expression increased SOX10 transcription and protein expression, albeit to lower levels compared with inhibition of ATF2 expression (Figure S4). Concomitant increase of MITF RNA and protein levels was also lower, compared with that seen upon inhibition of ATF2 expression. Notably, inhibition of both ATF2 and FOXD3 resulted in additive increase of SOX10 and MITF (Figure S4). These data suggest that FOXD3 may also contribute to negative regulation of MITF in melanocytes, independent of ATF2. Since inhibition of FOXD3 elicited a less pronounced effect compared with ATF2, and since the effect appeared ATF2-independent and furthermore did not appear to mediate similar changes in human melanoma cells (Figure S3b and data not shown), we focused on assessment of direct mechanisms underlying ATF2 effect on MITF transcription.

**Figure 5:**
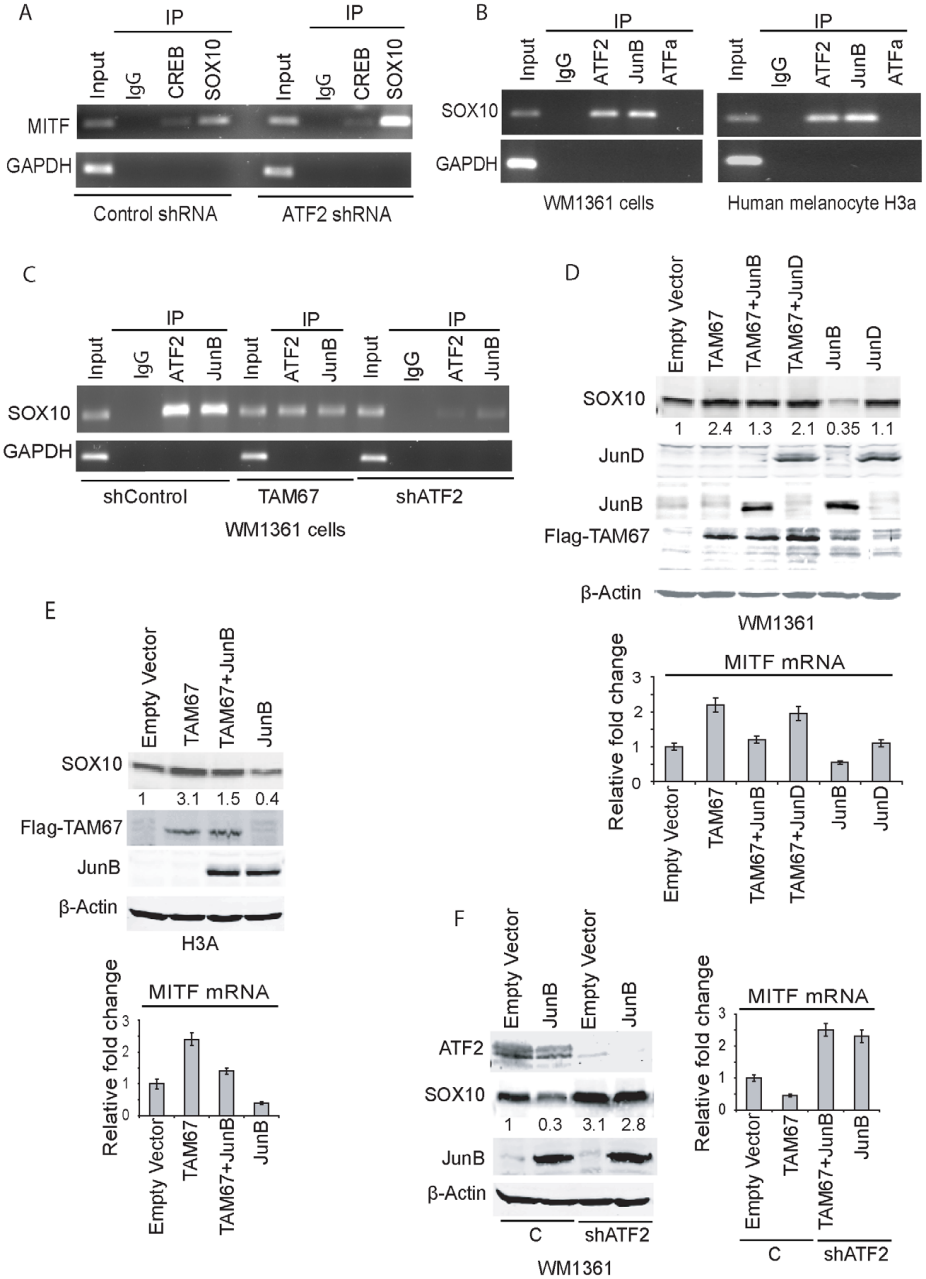
ATF2 and JunB negatively regulate SOX10 transcription, with concomitant effect on MITF. A. Chromatin immuno-precipitation were performed using antibodies to SOX10 or CREB (or rabbit IgG as control) in WM1361 melanoma cells expressing either control shRNA or ATF2 shRNA. The MITF promoter sequence spanning −300 to −120 (180bp containing SOX10 binding sites and a CRE site) was amplified using MITF-specific primers. GAPDH served as a control. B. ChIP was performed using the indicated antibodies followed by amplification of the SOX10 promoter sequence containing AP-1 binding sites (−4797 to −4791). GAPDH served as control. C. ChIP analysis was carried out using ATF2 or JunB antibodies in cells that express either shcontrol, shATF2 or Jun DN construct TAM67. D. Expression of JunB or JunD (3 µg) alone or in combination with TAM67 (2 µg) was performed in WM1361 melanoma cells and expression of SOX10 protein was monitored in westerns and quantified using the LICOR imaging system. Corresponding changes in level of MITF transcripts were assessed by qPCR (Lower panel). E. Experiment similar to shown in panel D was performed in the melanocytes H3A cell line. F. The effect of JunB on SOX10 expression was assessed in melanoma WM1361 cells expressing control or shATF2. The right panel shows the level of MITF transcripts quantified by qPCR.

To this end we first analyzed MITF promoter sequences for ATF2/CRE elements (Cyclic AMP response element), which can be targeted by ATF2, as well as sequences recognized by BRN2 and SOX10 using a luciferase reporter construct (MITF-Luc) [43]. Using either a wild-type (WT) construct or one in which the BRN2 site was mutated, we observed increased luciferase activity following inhibition of ATF2 transcription in WM1361 melanoma (Figure 4b), as well as in LU1205 and WM35 melanoma cells (data not shown). The relative increase in luciferase activity following ATF2 inhibition was equivalent in both constructs, suggesting that an ATF2 effect is not mediated by BRN2 (Figure 4b, left panel). Similarly, MITF transcriptional activities were altered to a similar degree following inactivation of the CRE element (Figure 4b, right panel), suggesting that ATF2 down-regulation of the MITF promoter is indirect. We therefore assessed whether SOX10, which positively regulates MITF and whose transcription markedly increases in melanocytes and melanoma cells in which ATF2 expression is inhibited (Figure 4a, S3b), may mediate ATF2 effect on MITF transcription. Analysis of a MITF-Luc construct harboring a mutant SOX10 binding site revealed that ATF2 inhibition no longer elicited increased MITF transcription in human melanocytes or in melanoma cells (Figure 4c). In agreement, inhibition of SOX10 expression by corresponding siRNA attenuated the increase in MITF transcription seen in shATF2-expressing human melanocytes (Figure 4d) or melanoma cells (Figure 4e). These results suggest that ATF2 regulation of MITF transcription is mediated by SOX10. In agreement, chromatin immunoprecipitation (ChIP) assays confirmed increased binding of SOX10 to the MITF promoter in melanoma cells expressing shATF2 (Figure 5a).

A putative response element for AP1 (which can serve as an ATF2 response element through ATF2 heterodimerization with JUN family members; [9]) has been identified in upstream regions of the Sox10 promoter [44]. We examined potential ATF2 binding to this element by ChIP and found that endogenous ATF2, but not ATFa, binds to that AP1 sequence (−4797–4791) in both human melanocytes and melanoma cells (Figure 5b). We next set to identify ATF2 heterodimeric partner, which could mediate negative regulation of SOX10 transcription. Among members of the JUN family implicated in transcriptional silencing is JunB. Thus, further assessment was performed to determine if JunB functions as an ATF2 heterodimerization partner to regulate SOX10 transcription through the AP1 site. ChIP analysis confirmed that JunB binds to the AP1 site found in SOX10 promoter sequences (Figure 5c). To confirm a possible role for JunB in regulating MITF transcription we asked whether expression of TAM67, a negative regulator of Jun family members, could attenuate the binding and transcriptional activities elicited by JunB. Expression of TAM67 indeed reduced the degree of ATF2 and JunB binding to the AP1 site on SOX10 promoter. Further, KD of ATF2 expression abolished binding of both ATF2 and JunB to the AP1 site on the SOX10 promoter (Figure 5c). These data confirm the presence of ATF2-JunB complex on Sox10 promoter and suggest that ATF2 recruits JunB for binding to the AP1 site on SOX10 promoter. To assess the role of JunB on SOX10 transcription we have monitored changes in Sox10 expression at the protein and RNA levels. Expression of TAM67 caused increased expression of SOX10 in both human melanoma (∼2 folds; Figure 5d) and melanocytes (∼3 folds; Figure 5e), indicating some relief of JunB inhibition. Co-expression of TAM67 with Jun B attenuated this increase, reducing the level of Sox10 expression to basal levels (Figure 5d, 5e). Over-expression of JunB, but not Jun D, effectively inhibited Sox10 expression in both the melanoma and melanocytes cells (Figure 5d, 5e). These data suggest that JunB mediates inhibition of Sox10 expression. To further reveal the role of ATF2 in this inhibition, we assessed the effect of JunB on Sox10 expression in cells expressing control shRNA or shATF2. While ectopic expression of JunB reduced the expression of Sox10 in control shRNA-expressing cells, such decrease was no longer seen in cells expressing shATF2 (Figure 5f). Collectively, these findings suggest that ATF2, in concert with JunB, is responsible for inhibition of Sox10 expression.

We next assessed the effect of ATF2 on SOX10 and MITF expression in 12 additional human melanoma cell lines. In all cases cells were infected with shATF2 and changes in SOX10 and MITF were monitored at the level of RNA. Notably, about 4/12 melanoma lines revealed increase in both SOX10 and MITF expression upon KD of ATF2 (Figure S5, Table 4). In contrast, 6/12 melanoma lines revealed decrease in MITF expression, of which 5 also shown decrease in SOX10 expression, pointing to positive regulation of SOX10 and MITF in these melanoma cells. In two out of the 12 melanoma lines ATF2 affected SOX10 but not MITF transcription (Figure S5). Overall, our cohort of 18 melanoma lines revealed that about 50% of the melanomas retained negative regulation of MITF by ATF2, as seen in the melanocytes (primary and cell lines) (Table 4).

To further assess whether ATF2 regulation of MITF is SOX10-dependent in melanocytes and melanoma cells, we coexpressed SOX10 in shATF2-expressing cells. As seen in earlier analysis, inhibition of ATF2 expression caused increase in MITF transcription in the human melanocytes and 4 melanoma cell lines, (WM1361, WM793, LU1205, WM35; Figure S6). Notably, the melanocytes and 2/4 melanoma cell lines revealed ATF2 effect on MITF expression is SOX10-dependent (WM1361, WM793; Figure S6). Two of the four melanoma cell lines did not reveal increased SOX10 expression, although they retained increased MITF expression, upon inhibition of ATF2 (Lu1205, WM35; Figure S6). These findings confirm that while in melanocytes, expression of SOX10 and MITF is negatively regulated by ATF2, this mechanism is conserved in approximately half of melanomas surveyed.

Along these lines, the two melanoma lines (MeWo and 501 Mel) that exhibit positive regulation of MITF by ATF2 also exhibited positive regulation of SOX10 by ATF2 (Figure S7). Inhibition of ATF2 expression reduced SOX10 and MITF RNA and protein levels (Figure S7a–c). In order to determine whether JunB lost its ability to elicit negative regulation of SOX10 and MITF in melanoma cells where ATF2 no longer inhibited SOX10 or MITF expression, we transfected those cell lines with TAM67 and JunB alone and in combination. In these cells, whereas TAM67 effectively attenuated Sox10 and MITF expression, JunB did not alter expression of these genes, suggesting that positive regulation of MITF and SOX10 by ATF2 depends on other members of the Jun family of transcription factors (Figure S7d). Conversely, TAM67 or JunB had no effect on melanoma cells in which ATF2 inhibits MITF independently of SOX10, suggesting that in these cases, ATF2 likely cooperates with transcription factors other than JunB to elicit negative regulation of SOX10 and MITF (Figure S7d). Consistent with this observation, ChIP assay confirmed ATF2 and CREB, but not JunB, binding to the Sox10 promoter in these cells (Figure S7e). These findings suggest that changes in ATF2 heterodimeric partner (from JunB to CREB) are likely to cause the switch from negative to positive regulation of SOX10, and in turn, MITF (see below). The possibility that altered expression of JunB may account for ATF2 positive or negative regulation of Sox10 and MITF were excluded, as no clear correlation between JunB expression and the ability of ATF2 to elicit negative regulation of Sox10/MITF were seen (Figure S7f).

Among response elements potentially required to upregulate MITF transcription is the CRE element, which is implicated in CREB-mediated upregulation of MITF transcription [45]. Although transcriptional activity from a CRE mutant MITF promoter was lower compared to the WT promoter (30%), it was no longer responsive to inhibition of ATF2 expression in the MeWo cells (Figure S8a). Pull-down assays using biotin-tagged MITF promoter sequences harboring the CRE identified ATF2 and CREB as CRE-bound proteins in MeWo melanoma cells (Figure S8b). In agreement, ChIP analysis confirmed occupancy of the CRE site on MITF promoter by ATF2 (Figure S8c). These findings are consistent with the fact that ATF2 heterodimerizes with CREB [9] and with a report that p38/MAPK14 (which phosphorylates ATF2) plays an important role in MITF transcription dependent on the CRE site [46]. These results establish that ATF2-dependent activation of MITF transcription in these melanoma cells is mediated through the CRE site, likely in cooperation with CREB. Notably, MeWo and 501Mel lines are known to express high MITF levels compared to other melanoma lines [41], [42], suggesting these cells harbor distinct mechanisms that preclude negative regulation of MITF by ATF2.

### Inhibition of MITF expression rescues focus formation on soft agar in shATF2-expressing melanocytes

**Figure 6:**
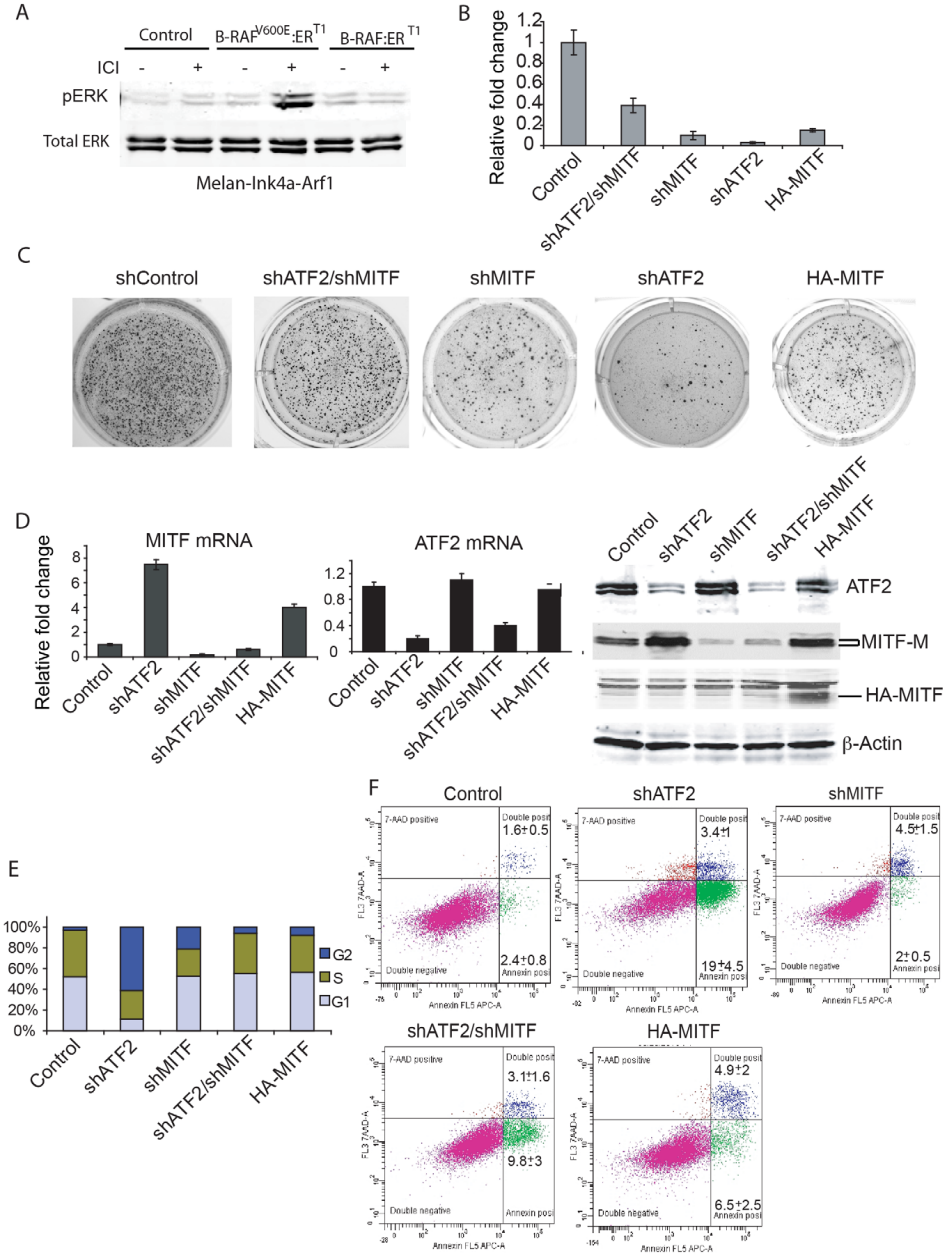
MITF down-regulation partially rescues colony formation by BRAF^V600E^-expressing mouse melanocytes with inhibited ATF2 expression. A. *Melan-Ink4a-Arf1* cells were stably transduced with wild type BRAF or BRAF^V600E^, followed by treatment with ICI 182780 (ICI, 200 nM) to induce BRAF expression. Western analysis was carried out to assess ERK activation using pERK and ERK antibodies. B. Mouse melanocytes stably expressing mutant B-RAF were infected with lentiviral vectors carrying shRNA for ATF2 or MITF or both. Cells were plated (5000 per well) on soft agar and assessed for the ability to form colonies after 21 days using P-Iodonitrotetrazolium Violet staining. Colonies were counted in triplicate wells per experiment, and experiments were reproduced twice. Means +SD are shown. C. Shown are representative images for quantification depicted in panel B. D. qPCR was performed for MITF (*left panel*) and ATF2 (*middle panel*) transcripts using RNA from cells used in panels B/C. Western analysis was performed on lysates obtained from cells used for colony formation with the indicated antibodies (*right panel*). E. Mouse melanocytes analyzed for colony formation were stained with BrdU. The cell cycle phase was analyzed using the Mod Fit LT v.2 program. The percentage of cells in G1, S and G2 is shown in the graph. F. Melanocytes shown in Panels A–E were also stained with Annexin V (early apoptosis) and 7-AAD (apoptosis and necrosis). The plot presented reveals Annexin-APC staining on the x-axis and 7-AAD staining on the y-axis. Mean +/− SD are calculated based on triplicate analyses.

To determine whether the contribution of ATF2 to melanocyte transformation and development is MITF-dependent, we assessed melanocytes' ability to grow and form colonies in soft agar, which is indicative of their transformed potential. Expression of mutant BRAF^V600E^ in immortal melanocytes is reportedly sufficient for growth on soft agar [47]. Thus we infected *melan-Ink4a-Arf1* melanocytes with mutant BRAF (Figure 6a) and confirmed their ability to form colonies in soft agar. Mutant BRAF expression effectively caused formation of about 1000 colonies per 5000 cells (Figure 6b, 6c). In contrast, melanocytes infected with BRAF^600E^ and with shATF2 formed on average about 20 colonies, indicative of loss of tumorigenicity (Figure 6b, 6c, 6d) and consistent with our initial observation that the number of melanoma tumors significantly decreases in the absence of transcriptionally functional ATF2 (Tables 1–2). To determine the importance of MITF at this early stage of melanocyte transformation we inhibited MITF expression (using shRNA) in melanocytes expressing mutant BRAF alone or mutant BRAF+shATF2. Significantly, inhibition of MITF expression decreased the number of BRAF-induced foci (from 1000 to about 100 per well). Over-expression of MITF in BRAF-expressing melanocytes also inhibited focus formation, to a degree similar to that seen following inhibition of MITF expression (Figure 6b, 6c, 6d). This observation implies that effective inhibition or overexpression of MITF attenuates melanocyte transformation, consistent with previous reports (52). Remarkably, inhibition of MITF expression in melanocytes expressing both mutant BRAF and shATF2 rescued, at least partially, melanocytes' ability to form foci on soft agar (400 compared with 20 seen in shATF2 cells; Figure 6b, 6c, 6d). These findings suggest that inhibition of MITF expression in melanocytes lacking ATF2 expression can promote transformation. That MITF inhibition in melanocytes expressing ATF2 WT can attenuate their ability to form foci on soft agar is attributable to the relative expression of MITF RNA and protein in each condition (Figure 6d). MITF expression levels in ATF2 KD cells increased 7.5-fold compared with control BRAF-expressing melanocytes. Inhibition of MITF expression in ATF2 KD melanocytes reduced MITF expression 2.5-fold relative to controls, whereas MITF KD alone resulted in lower MITF expression (5-fold; Figure 6d). Thus, complete abrogation of MITF expression attenuates melanocyte transformation, whereas low to moderate levels of MITF expression are sufficient to promote growth on soft agar. Higher MITF expression levels, as seen in ATF2 KD cells, result in a total loss of melanocytes' ability to form foci on soft agar. These findings are in line with the proposed rheostat model in which medium levels of MITF are optimal for growth and melanoma development [48] and in agreement with our observations in a mouse melanoma model.

We next assessed whether inhibition of melanocyte growth on soft agar by altered ATF2 and/or MITF expression can be attributed to decreased proliferation or increased apoptosis. Inhibition of ATF2 expression caused notable accumulation of cells in G2 (60%), with significant cell death induction (22%) compared to controls (4%), (Figure 6e, 6f). Interestingly, such altered cell cycle distribution and cell death rate were associated with a significant increase in MITF protein levels (Figure 6d). In contrast, inhibition of MITF expression did not significantly induce cell death (6.5%) but resulted in fewer cells in G2/M-phase and more cells in G1, compared with inhibition of ATF2 alone. These observations suggest that MITF inhibition is sufficient to reduce the rate of cell cycle progression through G2/M phase and that inhibited growth of BRAF^600E^-expressing melanocytes on soft agar may be attributed to abrogation of distinct cell cycle-regulatory mechanisms. Combined inhibition of ATF2 and MITF restored cell cycle distribution to that seen in control melanocytes, and reduced cell death from 22.4% to 12.9%. Of interest, MITF overexpression promoted a similar degree of cell death (11.4%) without altering cell cycle distribution, similar to combined inhibition of ATF2 and MITF (Figure 6e, 6f). Together, these observations suggest that simultaneous inhibition of ATF2 and MITF averts cell cycle abrogation induced when expression of either of these factors is perturbed individually, further substantiating regulation of MITF by ATF2.

### Low nuclear MITF expression in melanoma tumors that exhibit strong nuclear ATF2 expression is associated with poor prognosis

**Figure 7:**
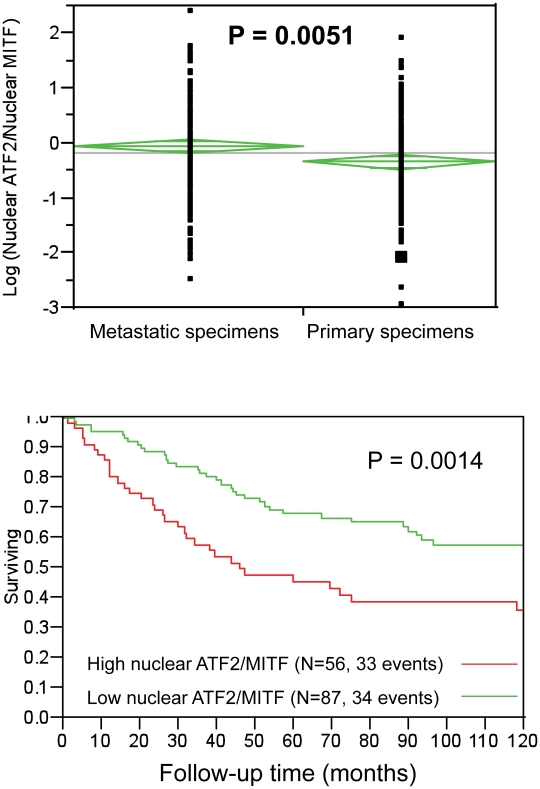
Analysis of nuclear ATF2 and MITF in melanoma specimens using quantitative immunofluorescence. A. ATF2/MITF expression ratios tended to be higher in metastatic than in primary specimens. B. In the primary specimen cohort, high ATF2/MITF ratios were associated with decreased 10-year disease specific survival. Quantification was performed by AQUA, which provides continuous output scores. In the absence of underlying cut-point justification, nuclear ATF2/MITF ratios were randomly binarized by the median ratio for all specimens (primary and metastatic). Results are shown for the primary cohort only, as no association was found between survival and ATF2/MITF ratios in the metastatic cases. The number of cases and events (deaths) for high or low ATF2/MITF expression ratios is shown.

The availability of a melanoma TMA, consisting of over 500 melanoma samples and in which expression of both ATF2 and MITF in the same tumors had been measured enabled us to assess possible associations between ATF2 and MITF and their correlation with survival and other clinical and pathological factors. Our earlier studies revealed that ATF2 subcellular localization in tumors is significantly correlated with prognosis: nuclear localization, reflecting constitutively active ATF2, was associated with metastasizing tumors and poor outcome [7]. Here we quantitated immunofluorescent staining of TMAs for MITF and ATF2 by employing our automated, quantitative (AQUA) method. To normalize ATF2 and MITF levels, expression of each of the two proteins in individual patients was divided by the median expression level of the respective protein in all patients, and the nuclear ATF2/MITF ratio was calculated and log-transformed. By ANOVA analysis, the ratio was higher in metastatic than in primary specimens (*t* value = 2.823, P = 0.0051), as shown in Figure 7a. No association was found between nuclear ATF2/MITF ratio and disease-specific survival among patients with metastatic melanoma (not shown). Significantly, a high nuclear ATF2/MITF ratio in primary melanoma specimens was associated with decreased 10-year disease-specific survival (P = 0.0014; Figure 7b). On Cox multivariable analysis, this association with survival was independent of patient age, Breslow thickness or the presence or absence of ulceration (data not shown). Nuclear ATF2 alone in primary specimens was associated with poor survival, but to a lesser degree than the ratio of nuclear ATF2/MITF (P = 0.0118 for ATF2 as a single discriminator versus P = 0.0014 for the ratio of nuclear ATF2/MITF). Nuclear MITF as a single discriminator was not a significant predictor of survival (P = 0.185), as was reported previously using immunohistochemistry [49]. These observations suggest that active (nuclear) ATF2 in melanoma can suppress MITF expression, and that this phenomenon is associated with poor prognosis.

## Discussion

Identifying mechanisms underlying early phases of melanocyte transformation and melanoma development is central to understanding the etiology of this devastating tumor, as well as for developing novel treatment approaches. Previous studies indicate the presence of mutant BRAF in melanocytic lesions, as well as its effect on pigment gene expression [6], [50], [51]. The present study enhances our understanding of early events contributing to melanoma development. We demonstrate that loss of a transcriptionally active form of ATF2 in melanocytes inhibits melanoma development in an Nras/Ink4a model. Our quest to understand mechanisms underlying ATF2 activity in this process led us to identify an important role for ATF2 regulation of MITF, an important regulator of melanocyte biogenesis and a factor implicated in melanoma progression [49]. Surprisingly, ATF2 negatively regulated MITF expression in mouse and human melanocytes, suggesting that ATF2 transcriptional activities limit MITF expression. We demonstrate that such negative regulation is elicited through downregulation of SOX10 by ATF2, in cooperation with JunB. A putative AP1 response element has been identified in SOX10 promoter sequences and ChIP analysis of this domain showed ATF2 and JunB binding. Overexpression of JunB efficiently suppressed SOX10 expression in an ATF2-dependent manner and inhibition of Jun transcriptional activities phenocopied the effect of shATF2, suggesting that negative regulation of SOX10 by ATF2 is direct, and is mediated in cooperation with JunB.

Importantly, ATF2-dependent negative regulation of Sox10 and consequently of MITF seen in melanocytes, but only in about 50% of the 18 melanoma cell lines studied here. Correspondingly, JunB, which is required for ATF2-dependent inhibition of Sox10 transcription, is no longer found on the promoter of SOX10 in melanoma cells (i.e. 501Mel) that exhibit positive regulation by ATF2. Rather, CREB and ATF2 are found on SOX10 and MITF promoters, pointing to a switch in ATF2 heterodimeric partners to enable positive regulation of these genes. Notably, melanoma cell lines that exhibit positive regulation of SOX10 and MITF by ATF2, also show high basal levels of MITF expression [41], [42], suggesting that additional genetic or epigenetic changes distinguish these lines from melanocytes and the other melanoma lines in which ATF2 elicits negative regulation of MITF.

Notably, ATF2 control of MITF expression affected the ability of BRAF^600E^-expressing melanocytes to exhibit transformed phenotype in culture, monitored by their ability to grow on soft agar. Inhibition of ATF2 abolished soft agar growth of BRAF^600E^-expressing melanocytes, which was partially rescued upon KD of MITF. Interestingly, both the over expression or the KD of MITF resulted in inhibition of melanocytes ability to grow on soft agar, substantiating the notion that a fine balance of MITF expression must be maintained in order to ensure its contribution to cellular proliferation and transformation. We propose that excessively low or high MITF levels block melanocyte transformation, whereas intermediate levels allow transformation to occur. Overall, our observations demonstrate that ATF2 plays an important role in fine-tuning those levels and support the rheostat model proposed for MITF's role in melanoma development and progression [48]. Of importance, ATF2 and MITF affect the ability of BRAF^600E^-expressing melanocytes to grow on soft agar via distinct mechanisms. Whereas specific inhibition of ATF2 causes both accumulation of cells in G2 and induction of cell death, specific alteration of MITF protein levels—particularly depletion—significantly affects cell proliferation and inhibit growth on soft agar by non-lethally slowing cell cycle progression at G2/M. These observations are consistent with a report from Wellbrock and Marais [52], who showed that altered MITF expression inhibits melanocyte proliferation.

Importantly, inhibiting MITF expression in ATF2 KD melanocytes was sufficient to partially rescue melanocyte growth on soft agar. While supportive of our finding in the *Nras::Ink4a* mouse melanoma model, where expression of transcriptionally inactive ATF2 inhibits melanoma formation, these observations provide the foundation for a model in which ATF2 inhibition causes increased MITF levels and concomitant inhibition of melanocyte growth, possible induction of cell death and delayed development. The latter is suggested by IHC analysis of mouse skin from *ATF2^md^* mice, which shows notably reduced S100 staining indicative of delayed melanocyte development: *ATF2* KO melanocytes appear to represent anagen stage IV, whereas WT represent anagen stage VI. This delay was seen at the 4- but not the 14-day time point, suggesting that an ATF2 effect might be limited to a specific subpopulation or phase of melanocyte development. The early (4 day) time point is within the time frame that allows induction of melanoma development by UV-irradiation of *c-Met* or *H-Ras* mutant mice [53]. It is therefore plausible that timely control of MITF expression by ATF2 determines melanocyte susceptibility to transformation.

Our analysis of genes whose expression is altered by ATF2 KD in melanocytes identified a cluster of pigmentation genes, many reportedly regulated by MITF [6], [54]. Therefore, changes in TYRP1, DCT and SILVER expression could be attributed to altered MITF expression. However, initial analysis points to a more complex mechanism since (i) the degree of changes in expression of these genes was often greater than that seen for MITF and (ii) expression of some pigmentation genes was found to be independent of MITF in some melanoma and melanocyte cultures. Hence, further studies are required to address mechanisms underlying ATF2 regulation of these pigmentation genes and the significance of such regulation to melanocyte transformation and melanoma development. While our present studies focused on the ATF2-MITF axis, it is expected that additional ATF2-regulated genes contribute to melanoma development [12]. In agreement, our earlier studies using both human and mouse melanoma lines demonstrate that inhibition of ATF2 effectively inhibits tumorigenesis and blocks metastasis [22]–[26].

Important for ATF2 function is its subcellular localization. While findings presented here position ATF2 as an oncogene functioning in melanocyte transformation and melanoma development, earlier studies from our laboratory and others suggest that in keratinocytes and mammary glands, ATF2 elicits a tumor suppressor function [55], [56]. Of interest, assessing the localization of ATF2 in the melanoma cell lines studied here revealed that all express nuclear ATF2. Interestingly, in most cases the nuclear staining revealed a punctate staining, resembling the localization of ATF2 to DNA repair foci following DNA damage (Figure S9). A possible link between the presence of ATF2 in repair foci in most melanoma cells points to the possible presence of activated DNA damage response which may be associated with genomic instability [19], [20]—aspects that will be explored in future studies. Significantly, the appearance of nuclear ATF2 is correlated with poor prognosis in melanoma, whereas melanomas that exhibit cytosolic ATF2 exhibit a better survival. Notably, cytosolic ATF2 is primarily seen in non-malignant skin tumors [55]. Here we demonstrate that high nuclear ATF2/MITF ratios are associated with poor prognosis in primary melanomas, but not with metastatic melanomas. The latter finding attests for the important role ATF2 plays to control MITF expression in the early phase of melanocyte transformation and melanoma development.

Overall, using the mutant *Nras*/*Ink4a* melanoma model we provide genetic evidence for a central role for ATF2 in melanoma development. We demonstrate that in the absence of transcriptionally active ATF2, melanoma formation is largely inhibited. Furthermore, our data point to an unexpected role of ATF2 in fine-tuning of MITF transcription through regulation of its positive regulator SOX10. Mouse melanoma models and in vitro transformation studies indicate that this newly identified regulatory pathway is required for early phases of melanocyte transformation. Given that ATF2 affects activity of the oncogenes *N-Ras* (mouse model) and *BRAF* (melanocyte growth on soft agar); we expect that ATF2 play significant roles in melanomas that carry either of these mutations.

## Materials and Methods

### Ethics statement

Research involving human participants has been approved by the institutional review board at Yale University (where the TMA was prepared and analyzed). All animal work has been conducted according to relevant national and international guidelines in accordance with recommendations of the Weatherall report and approved by the IACUC committee at SBMRI.

### Animal treatment and tumor induction protocols

Mice bearing a conditional allele for mutant ATF2 in which the DNA binding domain and part of the leucine zipper domain were deleted, were generated as previously described [36], [55]. To study the function of ATF2 in melanocytes, we utilized the Cre-loxP system for disruption of the *ATF2* gene in melanocytes [37]. The *Tyr::Cre^ER^::Atf2^md^* mice and their littermate controls (WT) were of FVB/129P2/OlaHsd (TyrCre^ERT^ mice were FVB, ATF2^fl/fl^ were 129P2/OlaHsd) and N-Ras/Ink4a^−/−^ mice were C57Bl/6/129SvJ. For melanoma studies we have used *Tyr::Cre^ER^::Nras^Q61K^::Ink4a^−/−^* mice (developed at HMS by LC) following their cross with the *Tyr::Cre^ER^::Atf2^md^* mice.

### Immunohistochemistry

Skin specimens were fixed in neutral buffered formalin solution and processed for paraffin embedding. Skin sections (5 µm in thickness) were prepared and deparaffinized using xylene. For MITF, DCT and S100 immunostaining, tissue sections were incubated in DAKO antigen retrieval solution, for 20 min in a boiling bath, followed by treatment with 3% hydrogen peroxide for 20 min. Antibodies against MITF (1∶100 from Sigma), DCT (1∶500, kind gift from Dr. Vincent Hearing) and S100 (1∶100, DAKOCytomation; Carpinteria, CA) were allowed to react with tissue sections at 4°C overnight. Biotinylated anti-rabbit IgG was allowed to react for 30 min at room temperature and diaminobenzidineor Nova Red were used for the color reaction. Hematoxylin was used for counterstaining. The control sections were treated with normal mouse serum or normal rabbit serum instead of each antibody.

### Cell culture

Immortalized human melanocytes Hermes 3A which has hTERT (puro) and CDK4 (neo) expression [57] were grown in RPMI 1640 medium containing Fetal Bovine Serum (FBS, 10%), 12-O-tetradecanoyl-phorbol-13-acetate (TPA, 200 nM, Sigma, St. Louis, MO), Cholera toxin (200 pM, Sigma), human stem cell factor (10 ng/ml, R&D systems, Minneapolis, MA), and endothelin 1 (10 nM, Bachem Bioscience Inc., Torrance, CA). Primary human melanocytes (NEM-LP; Invitrogen) were grown in medium 254 and HMGS (Cascade Biologics). Mouse melanocytes (*melan-Ink4a-Arf1*) were grown as for immortalized human melanocytes excluding human stem cell factor and endothelin. Melanoma cell lines were grown in DMEM medium supplemented with 10% FBS and penicillin/streptomycin (P/S; Cellgro). Melanoma cell lines used in this study LU1205, WM793, 501MEL, WM35, WM1361, MeWO (kind gift from Meenhard Herlyn), UACC903 were maintained in DMEM medium supplemented with 10% FBS and Penicillin/Streptomycin. Melanoma cell lines SbCl2, WM9, WM4, WM1650, A2068, WM1366, WM3629, WM1552, SKMEL2, SKMEL5, and SKMEL8 were maintained in RPMI medium supplemented with 10% FBS and Penicillin/Streptomycin. Primary melanocytes cultures were prepared from mice carrying the *Atf2* WT or mutant genotypes and *N-Ras/Ink4a^−/−^* as follows. Dorsal-lateral skin was removed from one day-old pups, disinfected with 70% ethanol for 1 min and then washed at least twice with sterile PBS. The skin was submerged in 1× Trypsin/EDTA overnight at 4°C and next day, the skin was placed in a Petri dish with mouse melanocyte culture medium (described below). The epidermis and sheared tissue was removed and discarded with forceps. The tissue was transferred to 15 ml centrifuge tubes and vortexed vigorously until solution becomes cloudy (1–2 min). The cell suspension was transferred to tissue culture flasks. After 3 days, melanocyte growth medium containing 0.8 µg/ml geneticin (Sigma-Aldrich) was added to eliminate contaminating fibroblasts (melanocytes are resistant to such treatment). Geneticin-containing medium was removed and replaced with fresh media after 1 day. Media was changed twice a week. Primary mouse melanocytes were grown in F-12 media (Invitrogen) containing 20% L-15 media (Invitrogen), 4% of FBS and Horse serum (Invitrogen), Penicillin (100 units) and streptomycin (50 µg) antibiotics, db-cAMP (40 µM, Sigma-Aldrich), 12-O-tetradecanoyl-phorbol-13-acetate (TPA, 50 ng/ml, Sigma-Aldrich), alpha-Melanocyte stimulating hormone (α-MSH, 80 nM, Sigma-Aldrich), Fungizone (2.5 µg/ml, Sigma-Aldrich) and melanocyte growth supplement (Invitrogen). Primary melanocytes were treated with 4-OHT (10 µM) for 8h followed by addition of doxycycline (2 µg/ml) for 24h to inactivate ATF2 and induce expression of N-Ras.

### Constructs

ATF2-specific shRNA clones were obtained from Open Biosystems (catalog no. RHS4533). Five different shRNA were obtained and tested for their efficiency of KD. Clone TRCN0000013714 was more efficient in inhibiting ATF2 in human cell lines while clone TRCN0000013713 was more efficient for knocking down mouse ATF2. For subsequent experiments we used the respective shATF2 clone depending on human or mouse cell lines. We also tested 3 different clones for KD of ATF2 to rule out any off target effect (Data not shown). siRNA control (cat # 4611) and three SOX10-specific siRNA oligonucleotides were obtained from Ambion (cat # 4392420). Four FOXD3 specific siRNA were obtained from Dharmacon (Cat # J-009152-06 -07, -08, -09). These siRNAs were pooled together in equimolar ratio for transient transfection. An MITF specific shRNA, and MITF promoter luciferase constructs (WT and mutant CRE-Luc constructs) were obtained from Dr. David Fisher [58]. pGL3 vectors containing wild-type and BRN2-site-mutated MITF promoters were obtained from Dr. Colin Goding [34]. pGL3 vectors containing wild-type and SOX10-site-mutated MITF promoters were obtained from Dr. Michel Goossens [59]. Retroviral vectors encoding a fusion protein consisting of full length human BRAF and BRAF^V600E^ linked to the T1 form of the human estrogen receptor hormone-binding domain were generously provided by Dr. Martin McMahon [60]. SOX10 expression vector obtained from Dr. Alexey Terskikh, RSV-JunB, RSV-JunD were obtained from Dr. Michael Karin and pBabe-Flag-TAM67 from Dr. Michael Birrer.

### Antibodies and immunoblotting

Antibodies against SOX10 and CREB (sc-1734 and sc-186 respectively) were from Santa Cruz Biotechnologies; antibodies against ATF2, pERK and ERK (catalogue # 9226, 4337 and 4695 respectively) were obtained from Cell Signaling; antibodies against MITF (C5) were purchased from Cell Lab vision. Protein extract (40–60 µg) preparation and western blot analysis were done as described previously [8]. Specific bands were detected using fluorescent-labeled secondary antibodies (Invitrogen, Carlsbad, CA) and analyzed using an Odyssey Infrared Scanner (Li-COR Biosciences). β-Actin antibody was used for monitoring loading.

### Immunofluorescence

Human melanoma and melanocytes were grown in coverslips, fixed (4% paraformaldehyde and 2% sucrose in 1×PBS), and then permeabilized and blocked (0.4% Triton X-100 and 2% BSA in 1×PBS) at room temperature. The cells were then washed (0.2% Triton X-100 and 0.2% BSA in 1×PBS) and incubated overnight at 4°C with monoclonal anti-rabbit antibody against ATF2 (20F1, 1∶100), followed by five washes and then subsequent incubation at room temperature for 2 h with anti-rabbit IgG (Invitrogen, 1∶300) and Phalloidin (Molecular Probes, 1∶1000). DNA was counterstained with 4,6-diamidino-2-phenylindole (DAPI; Vector Laboratories) containing mounting medium.

### Analysis of skin samples

Skin samples were collected from the backs of mice and immediately fixed with Z-fix, processed, and embedded in paraffin. Paraffin sections were routinely stained by H&E. Dewaxed tissue sections (4.0–5.0 µm) were immunostained using rabbit polyclonal antibodies to MITF (Sigma-Aldrich), S100 (S100B; DAKOCytomation; Carpinteria, CA), and DCT (αPEP8, kindly provided by Dr. Vincent Hearing). Application of the primary antibody was followed by incubation with goat anti-rabbit polymer-based EnVision-HRP-enzyme conjugate (DakoCytomation). DAB (DakoCytomation) or SG-Vector (Vector Lab, Inc.; Burlingame, CA) chromogens were applied, yielding brown (DAB) and black (SG) colors, respectively.

### Quantitative analysis of immunostaining

Quantitative analysis was performed as described previously [61]. Briefly, all slides were scanned at an absolute magnification of 400× [resolution of 0.25 µm/pixel (100,000 pix/in.)] using the Aperio ScanScope CS system (Aperio Technologies; Vista, CA). The acquired digital images representing whole tissue sections were analyzed applying the Spectrum Analysis algorithm package and ImageScope analysis software (version 9; Aperio Technologies, Inc.) to quantify IHC and histochemical stainings. These algorithms make use of a color deconvolution method [62] to separate stains. Algorithm parameters were set to achieve concordance with manual scoring on a number of high-power fields, including intensity thresholds for positivity and parameters that control cell segmentation using the nuclear algorithm.

### Microarray analysis

Primary melanocytes were treated with 4-OHT and Doxycycline before isolation of total RNA. 500 ng of total RNA was used for synthesis of biotin-labeled cRNA using an RNA amplification kit (Ambion). The biotinylated cRNA is labeled by incubation with streptavidin-Cy3 to generate probe for hybridization with the Mouse-6 Expression BeadChip (Illumina MOUSE-6_V1_1_11234304_A) that represents 46.6K mouse gene transcripts. We analyzed the BeadChips using the manufacturers BeadArray Reader and collected primary data using the supplied Scanner software. Data analysis was done as follows. First, expression intensities were calculated for each gene probed on the array for all hybridizations using illumina's BeadStudio 3.0 software. Second, intensity values were quality controlled and normalized: quality control was carried out by using the BeadStudio detection P-value set to <0.01 as a cutoff. This removed genes which were never detected in the arrays. All the arrays were then normalized using the cubic spline routine from the BeadStudio 3.0 software. This procedure accounted for any variation in hybridization intensity between the individual arrays. Finally, these normalized data were analyzed for differentially expressed genes. The groups of 2 biological and 2 technical replicates were described to the BeadStudio 3.0 software and significantly differentially expressed genes were determined on the basis of the difference changes in expression level (Illumina DiffScor>60 or DiffScore<−60) and expression difference p-value<0.01. Microarray data are available under accession number GSE23860.

### ShRNA infection and RNA interference

Human embryonic kidney 293T cells were transfected with corresponding retro- or lentiviral shRNA constructs (10 µg), Gag-pol (5 µg) and ENV expression vectors (10 µg) by calcium phosphate transfection into 10 cm plates and supernatant was collected after 48 hours to obtain viral particles. 2 million melanocytes and melanoma cells in 10 cm plates were infected with 5 ml of viral supernatant along with 5 ml of medium in the presence of 8 µg/ml polybrene. The virus was replaced with fresh media after 8 hours of infection. After two days, puromycin (1.5 µg/ml) was used to select cells for 3 days. For human and mouse melanocytes the media was changed to DMEM containing 10% FBS 24 h prior to harvesting cells. 50 nM duplexes of scrambled and SOX10- or FOXD3- specific siRNA were transfected into human melanocytes and WM1361 melanoma cells (2 million cells per transfection) by Nucleofection using Amaxa reagents (NHEM-Neo Nucleofector and Solution R respectively) for SOX10 or FOXD3 knock down. Over 90% of the cells transduced were able to resist drug selection, indicating efficient infection of the respective genes. GFP was also used to monitor efficiency of infection, confirming >90% GFP expression by fluorescence microscopy.

### Real-time quantitative reverse transcription–PCR (RT–PCR)

Quantitative PCR was performed as described earlier [8]. Total RNA was isolated using an RNeasy mini kit (Sigma, St. Louis, MO) and reverse transcribed using a high cDNA capacity reverse transcription kit (Applied Biosystems, Foster City, CA) following the manufacturer's instructions. Specific primers (Valuegene, San Diego, CA) used for PCR were as follows: Human ATF2, forward: tgtggccagcgttttaccaa, reverse: tgatgtgggctgtgcagttt., human MITF, forward: aaaccccaccaagtaccaca, reverse: acatggcaagctcaggac., human SOX10, forward: caa gtaccagcccaggcggc, reverse: gggtgccggtggtccaagtg., human FOXD3, forward: gcgacgggctggaagag, reverse: gctgtccgtgatggggtgcc., human PAX3, forward: ggaactggagcgtgcttttg, reverse: ggcggttgctaaaccagac., human BRN2, forward: gaaagagcgagcgaggaga, reverse: caggctgtagtggttagacg., mouse MITF, forward: agatttgagatgctcatcccc, reverse: gatgcgtgatgtcatactgga, mouse TYRP1, forward: ccctagcctatatctccctttt, reverse: taccatcgtggggataatggc., mouse DCT, forward: gtcctccactcttttacagacg, reverse: attcggttgtgaccaatgggt, mouse Silver, forward: tgacggtggaccctgcccat, reverse: agctttgcgtggcccgtagc. The reaction mixture was denatured at 95°C for 10 min, followed by 40 cycles of 95°C for 15s, annealing at 60°C for 30s and extension at 72°C for 30s. Reactions were performed using the SYBR *Green* qPCR reagent (Invitrogen) and run on an MX3000P qPCR machine (Stratagene, La Jolla, CA). The specificity of the products was verified by melting curve analysis and agarose gels. The amount of the target transcript was related to that of a reference gene (Cyclophilin A for both human and mouse) by the Ct method. Each sample was assayed at least in triplicate and was reproduced at least three times.

### Chromatin immunoprecipitation

Chromatin immunoprecipitation was performed using the Magna-Chip (Upstate) according to the manufacturer's instructions. Control shRNA and ATF2 knocked down WM1361 cells (one 10 cm plate for each, 80% confluent) were fixed in 37% formaldehyde and sheared chromatin was immunoprecipitated and subjected to PCR for 32 cycles. The following primers corresponding to the MITF promoter, spanning the SOX10 binding site were used, forward: gcagtcggaagtggcag, reverse: caactcactgtcagatcaa. Antibodies against Sox10 and CREB (sc-1734 and sc-186 respectively) were from Santa Cruz Biotechnologies. IgG control, and glyceraldehyde-3-phosphate dehydrogenase oligonucleotides were provided by the kit. Antibody against ATF2 (sc-6233), JunB (sc-8051), JunD (sc-74) were obtained from Santa Cruz. Antibodies against ATFa were provided generated by Nic Jones. For Sox10 promoter, the following primers spanning AP-1 binding site were used; forward: cccagtgctggcctaatagc, reverse: cacccttgatatccccaagtga.

### Luciferase assays

MeWo, WM35, WM1361, Lu1205 cells in six-well plates were transiently transfected with 0.5 µg of reporter plasmid containing WT or CRE mutant, BRN2 mutant or SOX10 mutant MITF promoter and 0.1 µg of pSV-β-Galactosidase (Promega, San Luis Obispo, CA) using Lipofectamine 2000 reagent (Invitrogen). Human melanocytes (2 million) were transfected with 2 µg of reporter plasmid containing WT or SOX10 mutant MITF promoter and 0.3 µg of pSV-β-Galactosidase using Amaxa reagent (NHEM-Neo nucleofector kit, Lonza) according to the manufacturer's protocol. Cell lysates were prepared from cells after 48 h. Luciferase activity was measured using the Luciferase assay system (Promega) in a luminometer and normalized to β-galactosidase activity. The data were normalized to β-galactosidase and represent the mean and SD of assays performed in triplicate. All experiments were performed a minimum of 3 times.

### Colony formation assay

*Melan-Ink4a-Arf1* cells were transduced with a retroviral vector expressing BRAF^V600E^:ER^T1^ and selected with puromycin for 3 days. These cells were treated with 200 nM of estrogen receptor antagonist ICI 182780 (ICI, Tocris Bioscience) to induce expression of BRAF^V600E^. After one day, these cells were transduced with a lentiviral vector expressing either shATF2 or shMITF separately, or in combination. Colony formation was carried out as described by Franken *et al.* [63]. Briefly, 5,000 cells were plated into each well of a 6-well plate, and cells were grown in mouse melanocyte media containing ICI and puromycin (1.5 µg/ml) for 3 weeks until colonies became visible. The colonies were stained with P-Iodonitrotetrazolium Violet (1 mg/ml Sigma, St. Louis, MO). This experiment was performed in triplicate and reproduced 2 times.

### Mouse genotyping

Genomic DNA was isolated from tail tissue was subjected to PCR resulting in amplification of a 549 bp DNA fragment for *Atf2* floxed and a 485 bp DNA fragment for wild type mice. PCR conditions included one cycle at 95°C for 3 min; and 30 cycles of 94°C/30 sec, 55°C/30 sec and 72°C/1 min and one cycle at 72°C for 5 min. Primers used for PCR reactions were forward: caatccactgccatggcctt, reverse: tcagataaagccaagtcgaatctgg.

### Avidin-biotin DNA–protein binding assay

MeWo cells were left untreated or treated with 20 mJ/cm^2^ of UV-B for 1 h. The cells were lysed using lysis buffer containing 1% Triton-100 and incubated with 4 µg of biotin-labeled MITF promoter spanning the CRE site oligo (5′-gaaaaaaaagcatgacgtcaagccaggggg-3′) in the presence of poly-(dI-dC) (20 µg/ml) for 2h at 4°c. The oligo-bound proteins were captured using streptavidin-agarose (Invitrogen) for 1 h incubation, followed by extensive washes with washing buffer (20 mm HEPES, 150 mm NaCl, 20% glycerol, 0.5 mm EDTA, and 1% Triton-100) and analyzed using SDS-PAGE and western blots.

### BrdU, PI labeling, Annexin V staining

To evaluate the cell cycle index of *Melan-Ink4a-Arf1* cells stably overexpressing BRAF^V600E^:ER^T1^ alone or in combination with shRNA to the genes indicated in Results, cells were plated in media containing ICI and puromycin (1.5 µg/ml) at 2×10^6^ cells per 10 cm plate O/N. Cells were labeled with 10 µM of 5-bromo-2-deoxyuridine (BrdU; Sigma Chemical Co.), for an hour. Cells were then washed, fixed, and stained with anti-BrdU mAbs and propidium Iodide (BD Biosciences, San Jose, CA) according to the manufacturer's protocol, and analyzed on a BD FACSCanto machine. Cell cycle phase was analyzed using the Mod Fit LT v.2 program (Verity Software, Topsham, ME). In a separate experiment the cells were stained with Annexin V-APC and 7-AAD (BD Pharmingen, San Diego, CA) according to manufacturer's protocol, to enable analysis of early apoptosis and cell death.

### Hypoxia treatment

Cells were treated under hypoxia (1% O_2_) for indicated time points using a hypoxia chamber (In Vivo 400; Ruskin Technologies Ltd, Bridgend, UK).

### UV irradiation

Mice were treated with 4-Hydroxytamoxifen (25 mg/ml in DMSO) by swabbing the entire body (excluding the head) on days 1–3 after birth. On day 4 the pups were placed under UVB light source (FL-15E; 320 nm) and exposed to 20 µW/cm^2^ for 22 seconds. Ninety minutes after UVB treatment mice were sacrificed and entire skin was removed and processed.

### TMA and AQUA staining

Tissue microarrays were constructed as previously described [21]. The arrays included a series of 192 sequentially collected primary melanomas and 299 metastatic melanomas. Slides were stained for automated, quantitative analysis (AQUA) for ATF2 and MITF as previously published [49], [64]. The AQUA scores for the two markers were obtained from the AQUAmine database (www.tissuearray.org).

## Supporting Information

Figure S1 Representative staining of non-melanoma tumor developed in the *TyrCre^+^*::*Atf2^+/+^::Nras^Q61K^::Ink4a^−/−^* model.
(7.25 MB TIF)

Figure S2 A. ATF2 negatively regulates MITF in several melanoma lines. ATF2 was knocked down in WM793 cells (*left panel*), in WM35 cells (*middle panel*) and in WM1361 cells (*right panel*) (one 10 cm plate each, 50% confluent). Cells were lysed and Western blotting was carried out (50 µg/lane) with the indicated antibodies. *Lower panel*, RNA was extracted from the corresponding cell lines, and qPCR was carried out using MITF primers. Cyclophilin A was served as an internal control. B. ATF2 positively regulates MITF in 501-MEL melanoma cells. *Left panel*, ATF2 was knocked down in 501- Mel cells (one 10 cm plate each, 50% confluent). Cells were lysed and Western blotting was carried out (40 µg/lane) with the indicated antibodies. *Right panel*, RNA was extracted from the above samples and qPCR was carried out using MITF primers; Cyclophilin A served as an internal control.
(1.36 MB TIF)

Figure S3 A. ATF2 effect on Brn2 and Pax3 in human melanocytes and melanoma cells. Cells were infected with shRNA control or shATF2 and RNA prepared was used for qPCR analysis of Pax3 and Brn2 expression, relative t o cyclophilin used as control. B. Increased SOX10 mRNA expression in melanoma cells. ATF2 expression was inhibited in WM1361 cells and total mRNA was extracted. A qPCR was performed to quantify changes in the expression of the indicated genes. Cyclophilin A served as an internal control.
(0.93 MB TIF)

Figure S4 Effect of ATF2 and FoxD3 on Sox10 and MITF expression in human melanocytes. H3a human melanocytes cells were infected with Scrambled Control siRNA (siSC), shATF2, siFOXD3 or their combination. Protein or RNA were prepared 72h later and assessed in western and qPCR.
(1.16 MB TIF)

Figure S5 Analysis of ATF2 effect on SOX10 and MITF transcription in 12 melanoma cell lines. Melanoma cell lines indicated were infected with shATF2 and subjected to selection to enrich for ATF2 KD cells. RNA was prepared 4 days later and QPCR analysis was performed for ATF2 (upper panel), MITF (middle panel) and SOX10 (lower panel) transcripts. Data shown represent analysis of triplicate samples.
(1.70 MB TIF)

Figure S6 Altered MITF expression by ATF2 +/− SOX10 in human melanocytes and melanomas. Indicated cell lines were infected with shControl or shATF2, in the presence or absence of SOX10 overexpression, and proteins were prepared to determine changes in expression of SOX10. β-actin was used as loading control. Analysis of MITF and SOX10 transcript levels was carried out in the indicated cell lines using qPCR on RNA prepared from the cells that were infected with indicated vectors (C, C+SOX10, shATF2, shATF2+SOX10).
(2.59 MB TIF)

Figure S7 Effect of ATF2 on SOX10 and MITF in human melanoma cell lines exhibiting positive regulation by ATF2. A. Melanoma cell lines MeWo and 501Mel were infected with shControl or shATF2 and level of SOX10 protein was assessed. B, C. The effect of SOX10 expression on MITF (B) and on SOX10 (C) transcripts in MeWO (left graph) cells expressing control or shATF2 was determined using qPCR. D. Effect of DN Jun and JunB on SOX10 protein and MITF transcript levels. Indicated melanoma cells were transfected with TAM67 (DN Jun) and JUNB either alone or in combination as indicated. The cells were lysed and proteins and RNA were prepared. Western blotting was carried out with the indicated antibodies. QPCR analysis for MITF transcripts was performed (lower panel). E. Chromatin IP reveals loss of JunB binding to SOX10 promoter in melanoma cells in which MITF is positively regulated by ATF2. Melanoma cells were subjected to ChIP using the indicated antibodies followed by PCR of SOX10 promoter sequences harboring the AP1 response element. F. Endogenous level of JUNB in various melanoma cells. Cell lysates from indicated melanoma cells were subjected to Western blot analysis using JUNB antibody. β–actin was used as a loading control.
(1.06 MB TIF)

Figure S8 A. Transcriptional activity of MITF is dependent on ATF2 and a CRE element in specific melanoma cells that express high MITF levels. WT and mutant (CRE) forms of MITF-Luc constructs were used to assess the contribution of ATF2 to MITF transcription in MeWo cells. Luciferase assays were performed as detailed in Materials and Methods. B. ATF2 and CREB bind to a MITF CRE site. Human melanoma (MeWo) cells were treated with 20mJ/cm^2^ of UVB, and proteins prepared after 1h were incubated with dI/dT (20 µg/ml for 30 min at 4C) and then with a biotinylated annealed oligo (4 µg, overnight at 4°C) containing the CRE site and flanking sequences from the MITF promoter. Streptavidin beads were added to lysates and bound material was analyzed on immunoblots with the indicated antibodies. C. ChIP analysis shows ATF2 binding to the MITF CRE site in specific melanoma MeWo cells. Chromatin was prepared and precipitated using an ATF2 antibody and bound DNA was amplified using MITF specific primers that flank the CRE binding site and analyzed on 1.5% agarose gel. Rabbit IgG served as a negative control and CREB and ATF1 served as positive controls.
(1.34 MB TIF)

Figure S9 Subcellular localization of ATF2 in melanoma cell lines. Immunostaining was carried out in indicated melanoma and human melanocyte cells with ATF2 (green) antibody and Phalloidin (red). The cells were counter stained with DAPI for nuclear staining.
(6.98 MB TIF)
