# Supplementary figures and images for: A Role for ATF2 in Regulating MITF and Melanoma Development
Source: PLoS Genet. 2010 Dec 23;6(12):e1001258. doi: 10.1371/journal.pgen.1001258 (PMC3009656; doi:10.1371/journal.pgen.1001258)

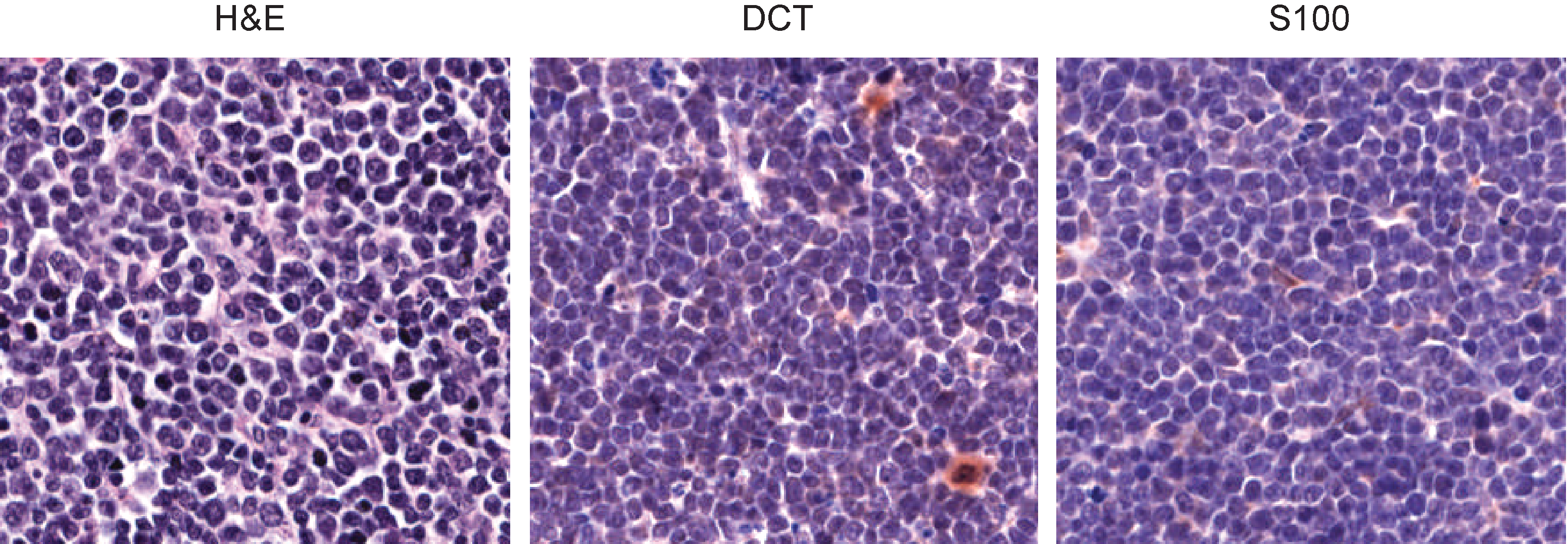

Supplement: Figure S1 — Representative staining of non-melanoma tumor developed in the TyrCre+::Atf2+/+::NrasQ61K::Ink4a−/− model. (7.25 MB TIF) [file pgen.1001258.s001.tif]

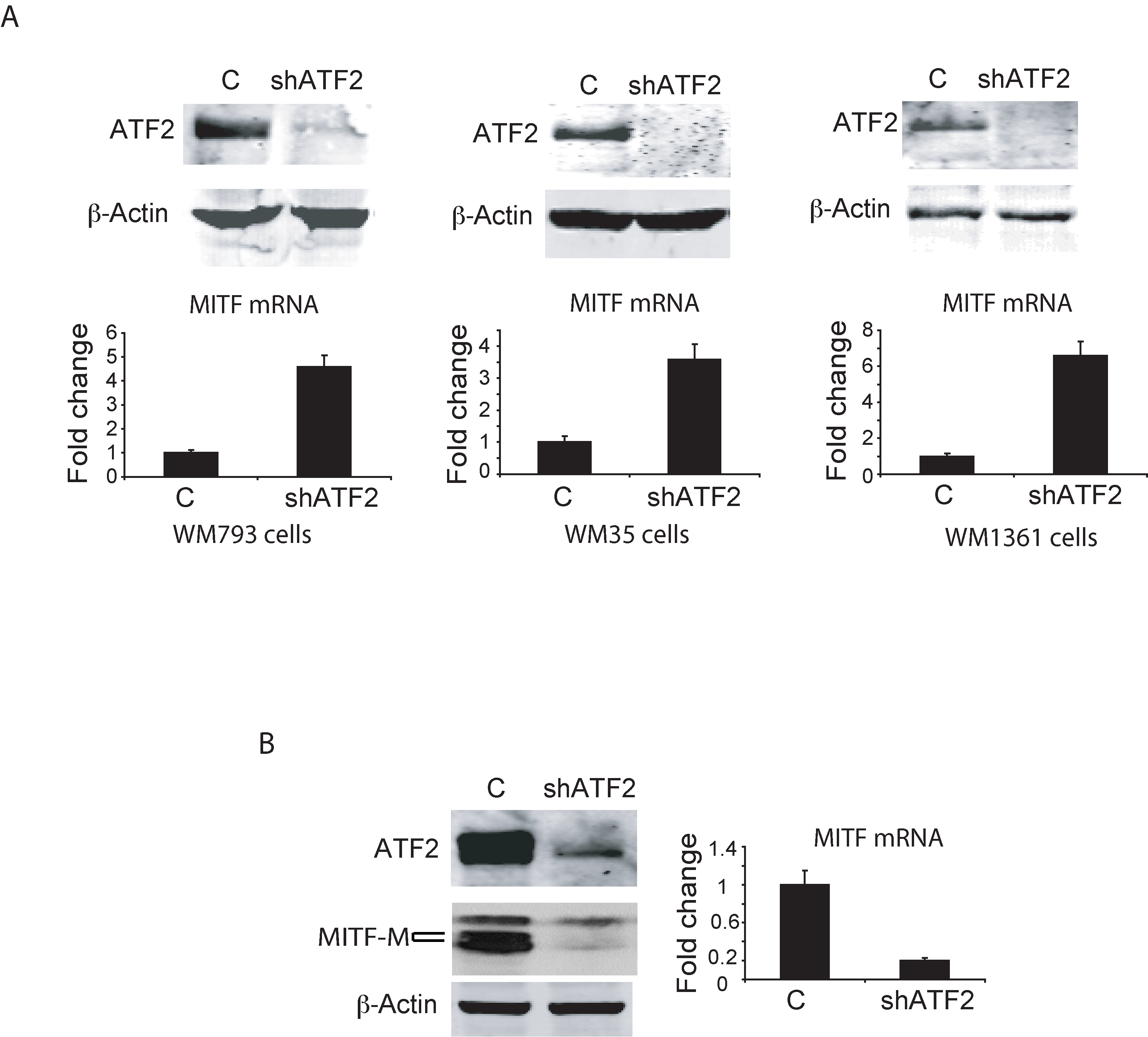

Supplement: Figure S2 — A. ATF2 negatively regulates MITF in several melanoma lines. ATF2 was knocked down in WM793 cells (left panel), in WM35 cells (middle panel) and in WM1361 cells (right panel) (one 10 cm plate each, 50% confluent). Cells were lysed and Western blotting was carried out (50 µg/lane) with the indicated antibodies. Lower panel, RNA was extracted from the corresponding cell lines, and qPCR was carried out using MITF primers. Cyclophilin A was served as an internal control. B. ATF2 positively regulates MITF in 501-MEL melanoma cells. Left panel, ATF2 was knocked down in 501- Mel cells (one 10 cm plate each, 50% confluent). Cells were lysed and Western blotting was carried out (40 µg/lane) with the indicated antibodies. Right panel, RNA was extracted from the above samples and qPCR was carried out using MITF primers; Cyclophilin A served as an internal control. (1.36 MB TIF) [file pgen.1001258.s002.tif]

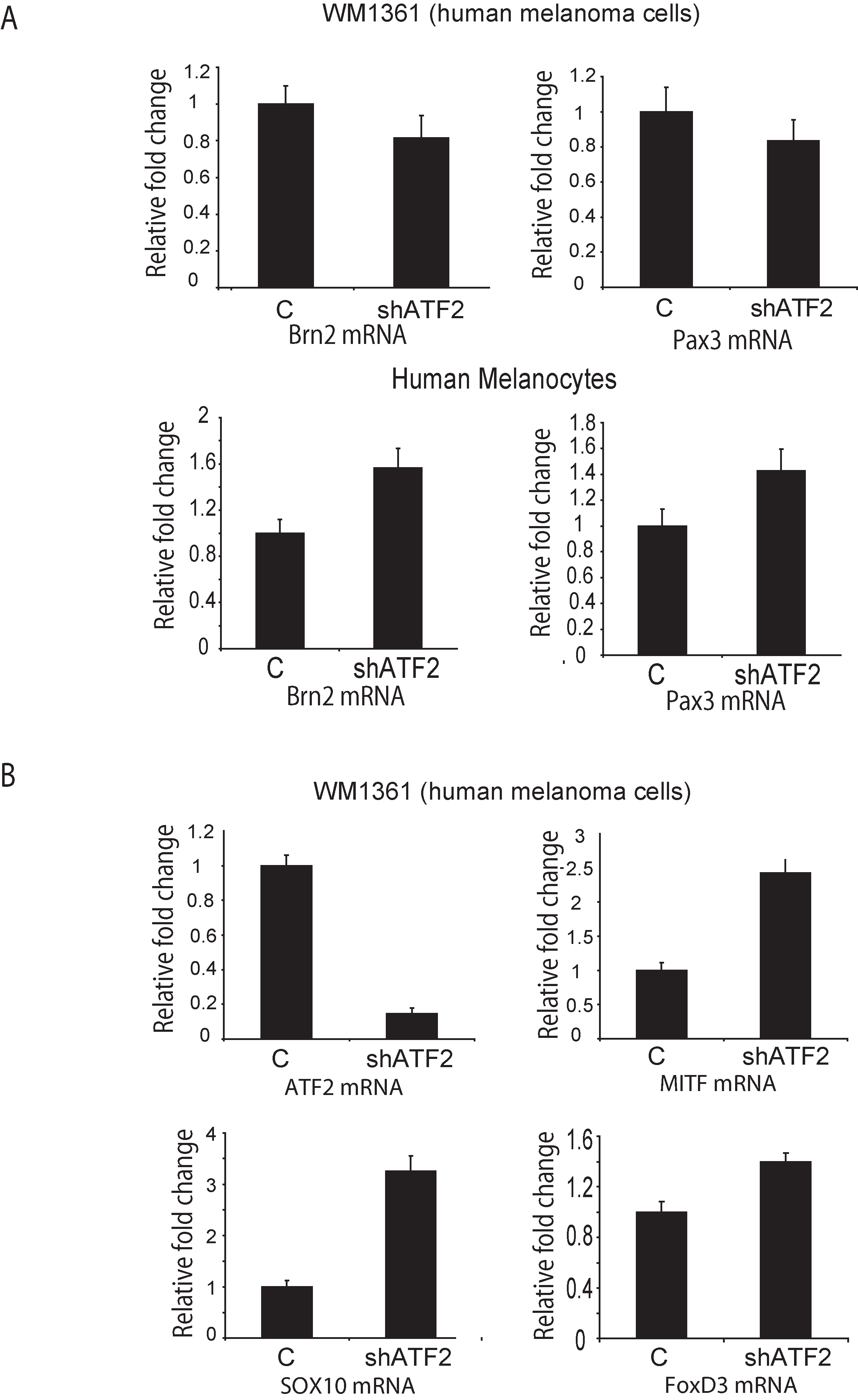

Supplement: Figure S3 — A. ATF2 effect on Brn2 and Pax3 in human melanocytes and melanoma cells. Cells were infected with shRNA control or shATF2 and RNA prepared was used for qPCR analysis of Pax3 and Brn2 expression, relative t o cyclophilin used as control. B. Increased SOX10 mRNA expression in melanoma cells. ATF2 expression was inhibited in WM1361 cells and total mRNA was extracted. A qPCR was performed to quantify changes in the expression of the indicated genes. Cyclophilin A served as an internal control. (0.93 MB TIF) [file pgen.1001258.s003.tif]

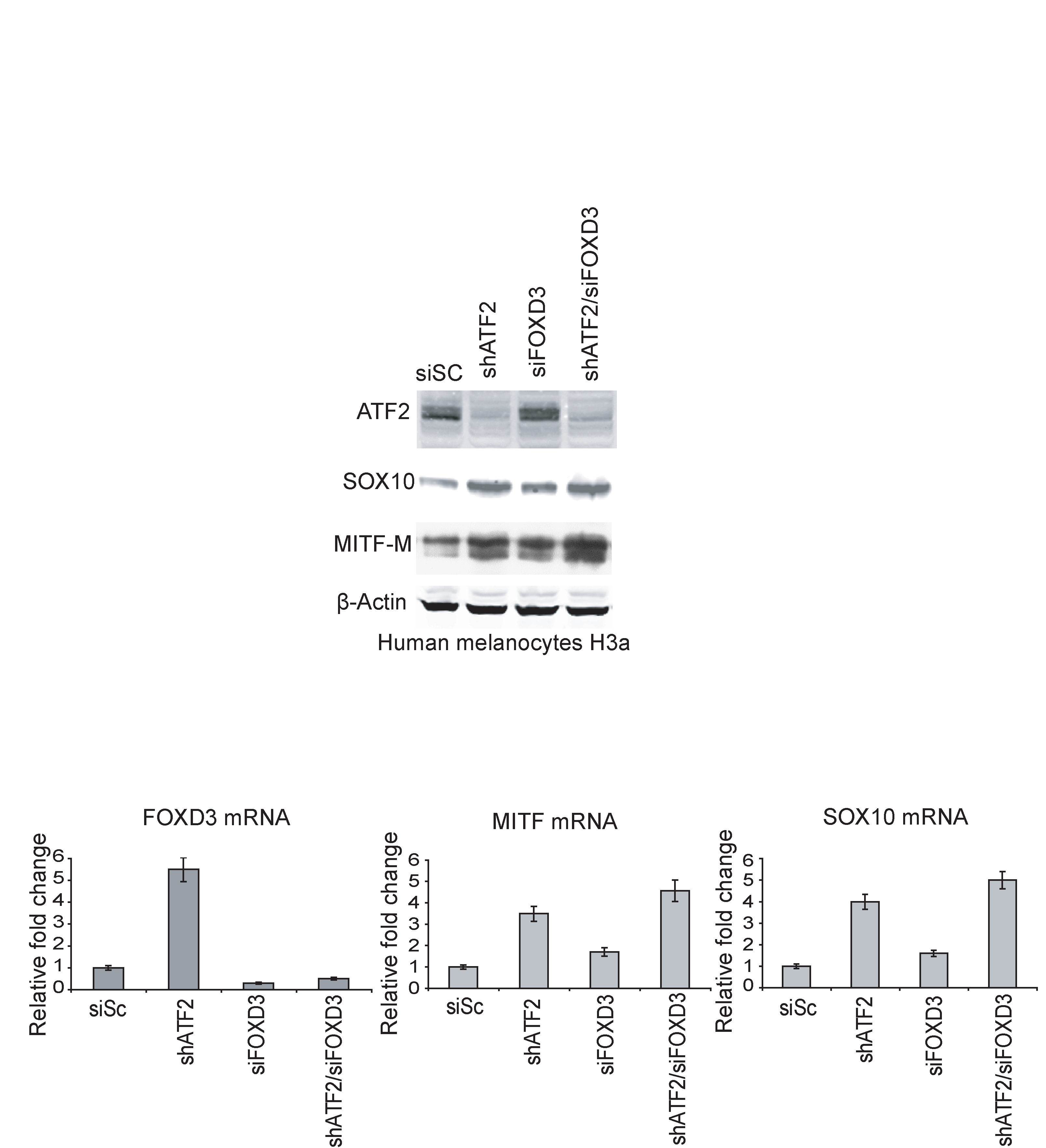

Supplement: Figure S4 — Effect of ATF2 and FoxD3 on Sox10 and MITF expression in human melanocytes. H3a human melanocytes cells were infected with Scrambled Control siRNA (siSC), shATF2, siFOXD3 or their combination. Protein or RNA were prepared 72h later and assessed in western and qPCR. (1.16 MB TIF) [file pgen.1001258.s004.tif]

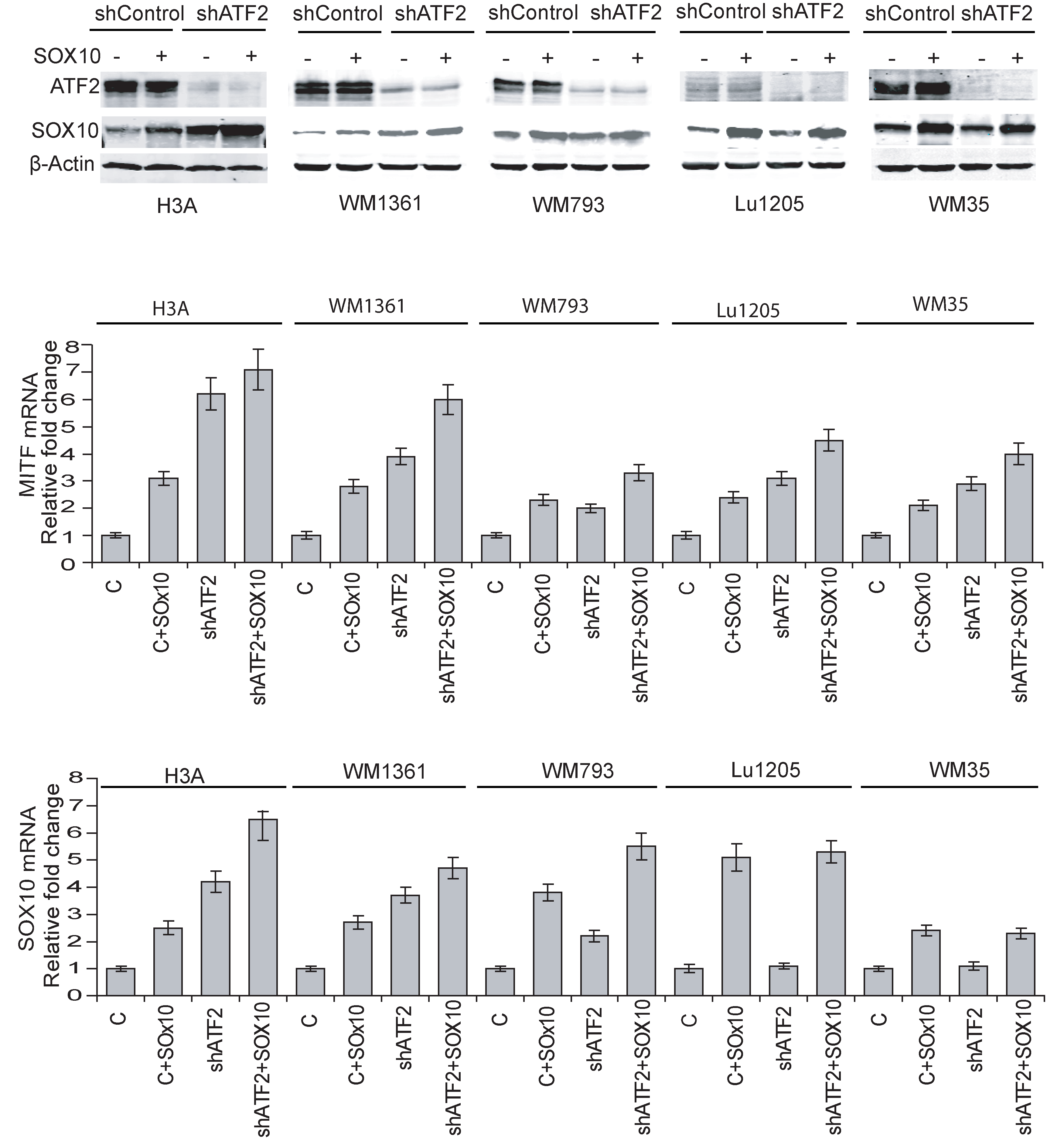

Supplement: Figure S5 — Analysis of ATF2 effect on SOX10 and MITF transcription in 12 melanoma cell lines. Melanoma cell lines indicated were infected with shATF2 and subjected to selection to enrich for ATF2 KD cells. RNA was prepared 4 days later and QPCR analysis was performed for ATF2 (upper panel), MITF (middle panel) and SOX10 (lower panel) transcripts. Data shown represent analysis of triplicate samples. (1.70 MB TIF) [file pgen.1001258.s005.tif]

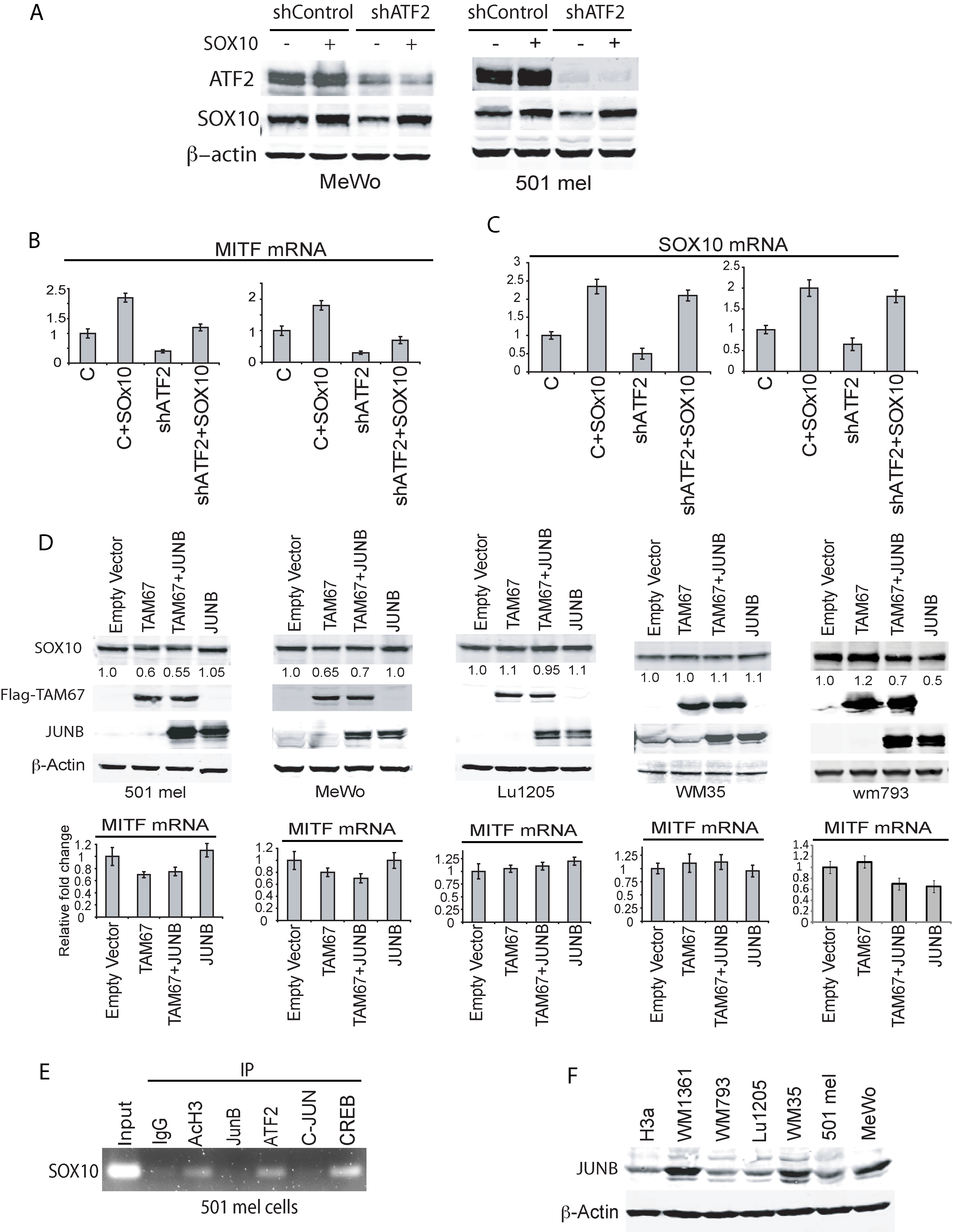

Supplement: Figure S6 — Altered MITF expression by ATF2 +/− SOX10 in human melanocytes and melanomas. Indicated cell lines were infected with shControl or shATF2, in the presence or absence of SOX10 overexpression, and proteins were prepared to determine changes in expression of SOX10. β-actin was used as loading control. Analysis of MITF and SOX10 transcript levels was carried out in the indicated cell lines using qPCR on RNA prepared from the cells that were infected with indicated vectors (C, C+SOX10, shATF2, shATF2+SOX10). (2.59 MB TIF) [file pgen.1001258.s006.tif]

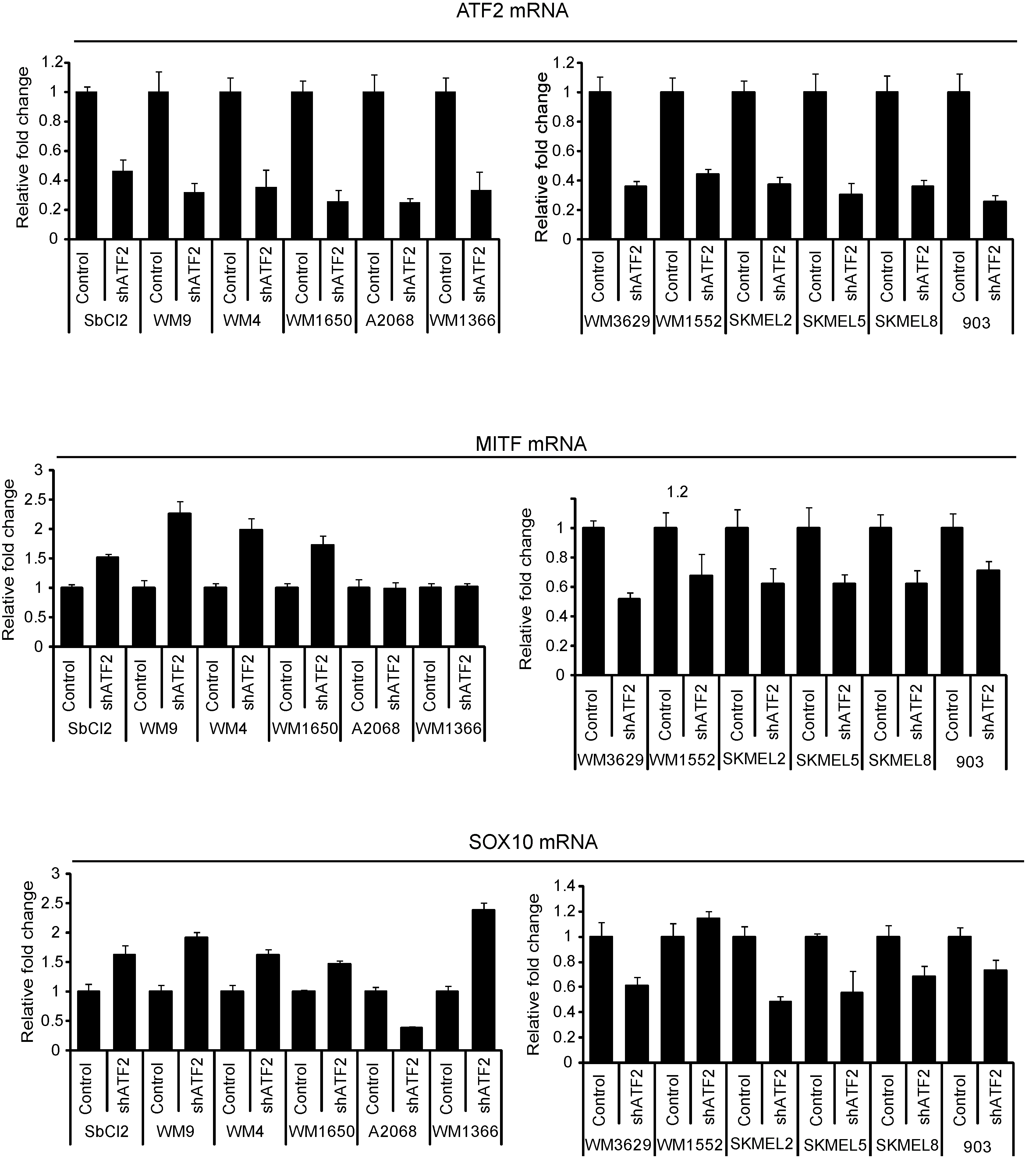

Supplement: Figure S7 — Effect of ATF2 on SOX10 and MITF in human melanoma cell lines exhibiting positive regulation by ATF2. A. Melanoma cell lines MeWo and 501Mel were infected with shControl or shATF2 and level of SOX10 protein was assessed. B, C. The effect of SOX10 expression on MITF (B) and on SOX10 (C) transcripts in MeWO (left graph) cells expressing control or shATF2 was determined using qPCR. D. Effect of DN Jun and JunB on SOX10 protein and MITF transcript levels. Indicated melanoma cells were transfected with TAM67 (DN Jun) and JUNB either alone or in combination as indicated. The cells were lysed and proteins and RNA were prepared. Western blotting was carried out with the indicated antibodies. QPCR analysis for MITF transcripts was performed (lower panel). E. Chromatin IP reveals loss of JunB binding to SOX10 promoter in melanoma cells in which MITF is positively regulated by ATF2. Melanoma cells were subjected to ChIP using the indicated antibodies followed by PCR of SOX10 promoter sequences harboring the AP1 response element. F. Endogenous level of JUNB in various melanoma cells. Cell lysates from indicated melanoma cells were subjected to Western blot analysis using JUNB antibody. β–actin was used as a loading control. (1.06 MB TIF) [file pgen.1001258.s007.tif]

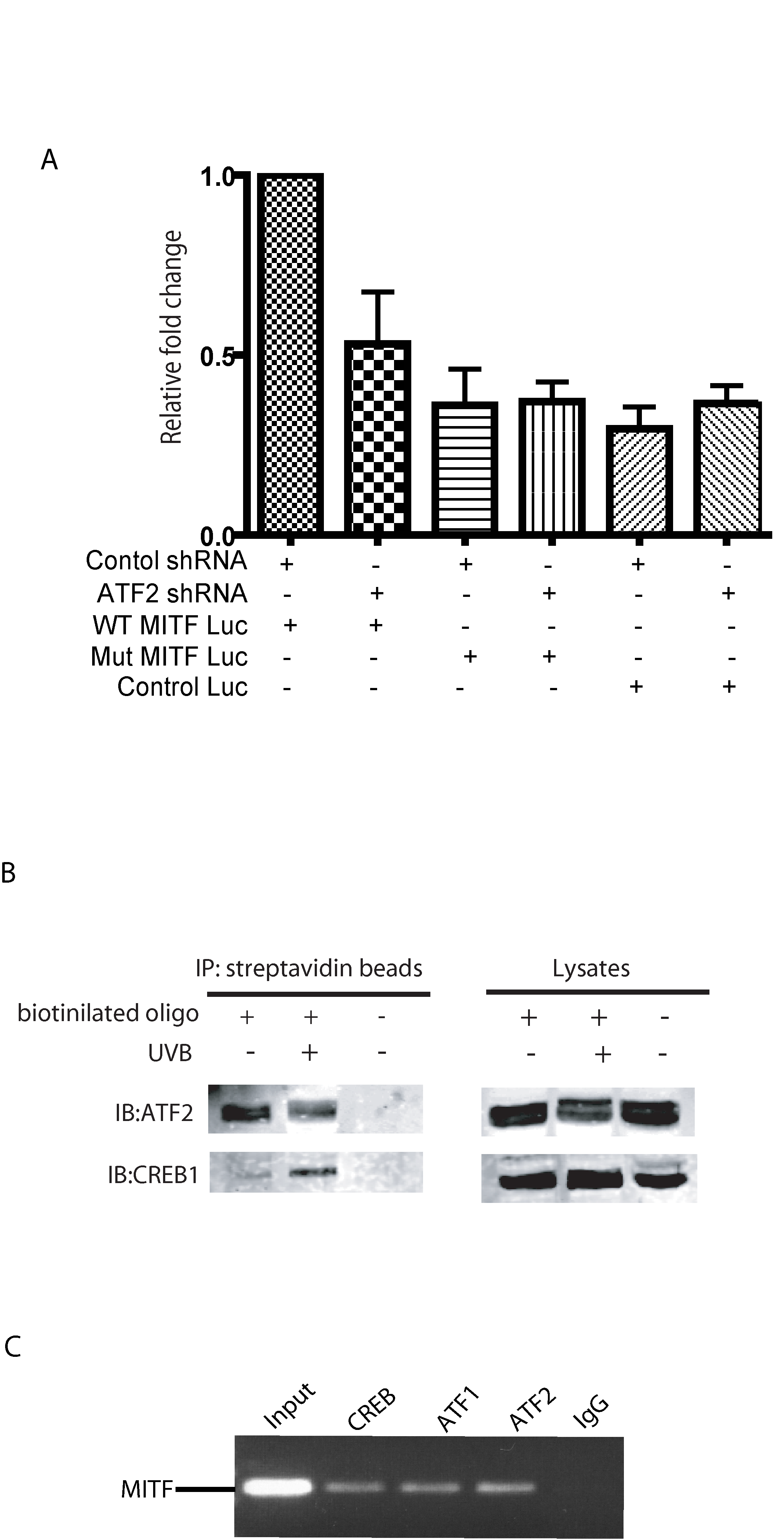

Supplement: Figure S8 — A. Transcriptional activity of MITF is dependent on ATF2 and a CRE element in specific melanoma cells that express high MITF levels. WT and mutant (CRE) forms of MITF-Luc constructs were used to assess the contribution of ATF2 to MITF transcription in MeWo cells. Luciferase assays were performed as detailed in Materials and Methods. B. ATF2 and CREB bind to a MITF CRE site. Human melanoma (MeWo) cells were treated with 20mJ/cm2 of UVB, and proteins prepared after 1h were incubated with dI/dT (20 µg/ml for 30 min at 4C) and then with a biotinylated annealed oligo (4 µg, overnight at 4°C) containing the CRE site and flanking sequences from the MITF promoter. Streptavidin beads were added to lysates and bound material was analyzed on immunoblots with the indicated antibodies. C. ChIP analysis shows ATF2 binding to the MITF CRE site in specific melanoma MeWo cells. Chromatin was prepared and precipitated using an ATF2 antibody and bound DNA was amplified using MITF specific primers that flank the CRE binding site and analyzed on 1.5% agarose gel. Rabbit IgG served as a negative control and CREB and ATF1 served as positive controls. (1.34 MB TIF) [file pgen.1001258.s008.tif]

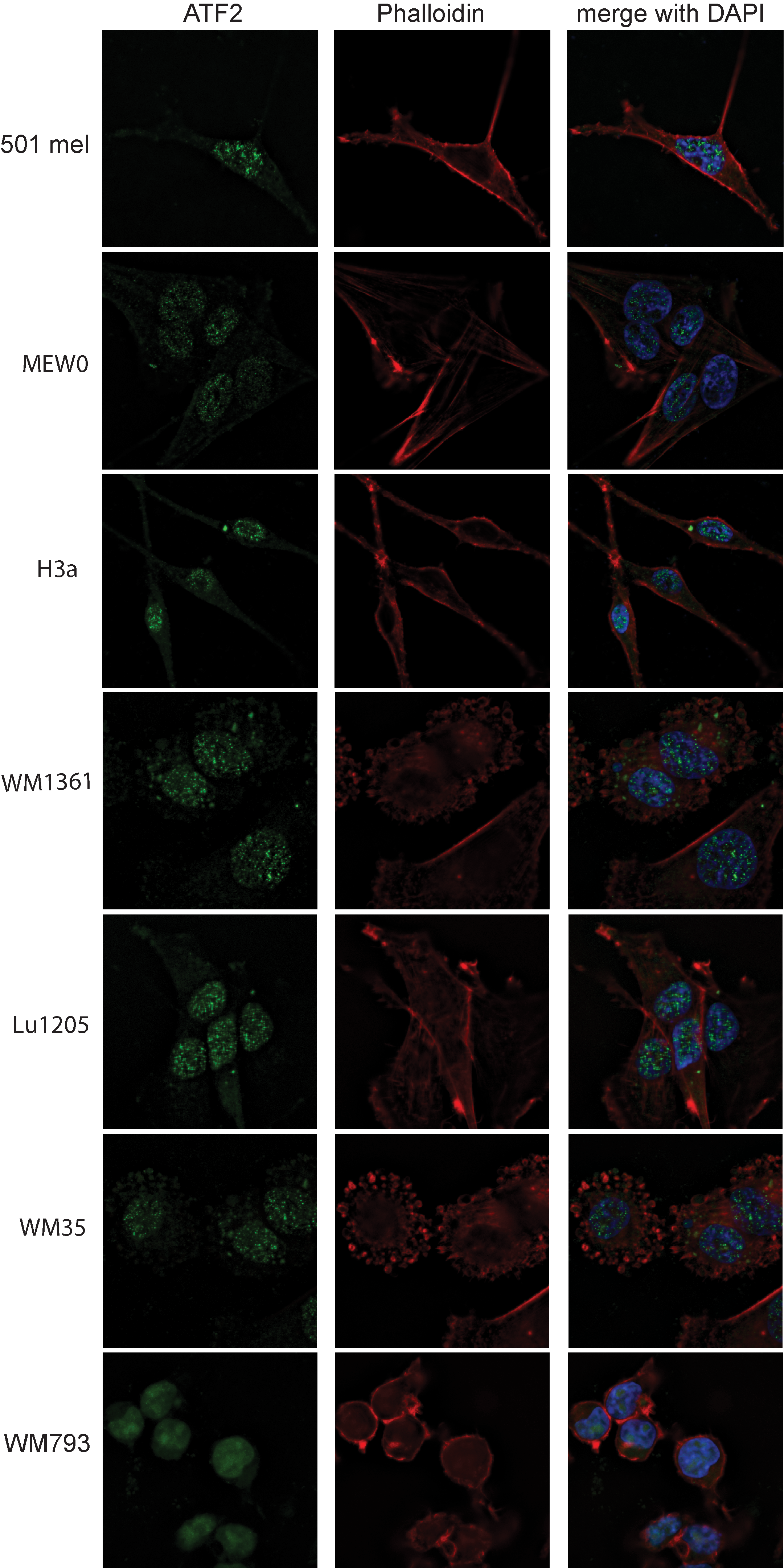

Supplement: Figure S9 — Subcellular localization of ATF2 in melanoma cell lines. Immunostaining was carried out in indicated melanoma and human melanocyte cells with ATF2 (green) antibody and Phalloidin (red). The cells were counter stained with DAPI for nuclear staining. (6.98 MB TIF) [file pgen.1001258.s009.tif]
